# VoPo leverages cellular heterogeneity for predictive modeling of single-cell data

**DOI:** 10.1038/s41467-020-17569-8

**Published:** 2020-07-27

**Authors:** Natalie Stanley, Ina A. Stelzer, Amy S. Tsai, Ramin Fallahzadeh, Edward Ganio, Martin Becker, Thanaphong Phongpreecha, Huda Nassar, Sajjad Ghaemi, Ivana Maric, Anthony Culos, Alan L. Chang, Maria Xenochristou, Xiaoyuan Han, Camilo Espinosa, Kristen Rumer, Laura Peterson, Franck Verdonk, Dyani Gaudilliere, Eileen Tsai, Dorien Feyaerts, Jakob Einhaus, Kazuo Ando, Ronald J. Wong, Gerlinde Obermoser, Gary M. Shaw, David K. Stevenson, Martin S. Angst, Brice Gaudilliere, Nima Aghaeepour

**Affiliations:** 1 Department of Anesthesiology, Perioperative and Pain Medicine, Stanford University, Stanford, USA; 2 Department of Biomedical Data Science, Stanford University, Stanford, USA; 3 Department of Pediatrics, Stanford University, Stanford, USA; 4 Department of Pathology, Stanford University, Stanford, USA; 5 Digital Technologies Research Centre, National Research Council Canada, Toronto, ON Canada; 6 Department of Plastic Surgery, Stanford University, Stanford, USA; 7 Center for Human Systems Immunology, Stanford University, Stanford, USA

**Keywords:** Cellular signalling networks, Applied immunology, Computer modelling

## Abstract

High-throughput single-cell analysis technologies produce an abundance of data that is critical for profiling the heterogeneity of cellular systems. We introduce VoPo (https://github.com/stanleyn/VoPo), a machine learning algorithm for predictive modeling and comprehensive visualization of the heterogeneity captured in large single-cell datasets. In three mass cytometry datasets, with the largest measuring hundreds of millions of cells over hundreds of samples, VoPo defines phenotypically and functionally homogeneous cell populations. VoPo further outperforms state-of-the-art machine learning algorithms in classification tasks, and identified immune-correlates of clinically-relevant parameters.

## Abstract (web summary)

Single-cell technologies are increasingly prominent in clinical applications, but predictive modelling with such data in large cohorts has remained computationally challenging. We developed a new algorithm, ‘VoPo’, for predictive modelling and visualization of single cell data for translational applications.

## Introduction

High-throughput single-cell analysis technologies offer key opportunities to investigate cellular heterogeneity at the levels of both gene and protein expression. However, uncovering a common set of phenotypically and functionally distinct cell populations from hundreds of millions of cells over hundreds of samples is a significant machine learning challenge that can be further complicated by batch effects and noisy measurements^1^.

Phenotypically-homogeneous cell populations can be identified through both unsupervised^1–6^ and supervised^7,8^ approaches. Single-cell data can further be linked to patient phenotypes through the use of classification-based methods^9,10^ or through rigorous statistical testing approaches^6,11^. CellCNN^9^ and CytoDX^10^ use single cells as input, rather than features extracted from computationally identified cell populations^3^. Independent from directly specifying a classification problem, though still making use of patient phenotypes, Diffcyt^6^ and Cydar^11^ belong to an additional class of algorithms that use patient labels for statistical testing to identify differentially-abundant or functionally-unique cell populations.

Unsupervised cell population discovery followed by feature extraction facilitates biological interpretation and predictive power in clinical applications^3^. Specifically, features engineered from computationally-identified cell populations can capture differences in cell-frequency or functional responsiveness between phenotypic classes. Many of these clustering-based algorithms are stochastic^2,4,5^ and lead to variable cell-to-cluster assignments across algorithm runs. Previous work has acknowledged the implications of stochasticity in the interpretation of automated cell-population discovery results^12^ and systematically analyzed the downstream effects of different sources of randomization in single-cell analyses^13^. However, to the best of our knowledge, no prior work has examined how stochasticity in cell-to-cluster assignments can lead to variation in engineered features and corresponding classification accuracy.

Motivated by the objective to efficiently engineer a set of interpretable and biologically-meaningful features from single-cell data, we examined the effects of clustering algorithm stochasticity on classification accuracy. Our analysis revealed that individual clustering solutions generated corresponding features that lead to variability in classification accuracy. In this work, we focused on how to leverage the diverse information content in each individual solution obtained from a stochastic clustering algorithm for improved prediction accuracy and interpretability in clinical settings.

VoPo (The name VoPo is an abbreviation for the concept of Vox Populi published by Sir Francis Galton in Nature more than a century ago, where he demonstrated that heterogeneity in estimations can be leveraged for more accurate predictions^14^) is a machine learning algorithm focused on cell-population discovery, robust predictive modeling, and visualization of the cellular correlates of patient phenotype. VoPo’s cell-population discovery method is readily scalable and enables the integration of a large number of samples without any downsampling, ensuring that rare cell types^15^ can be adequately identified. The clustering component of VoPo does not rely on an individual cell-to-population partition, but instead integrates multiple clustering solutions to predict patient phenotype. In doing so, VoPo increases classification accuracy and has less variability compared to individual solutions. VoPo further links these identified cell populations with additional clinical information to provide a comprehensive understanding of the cell types and signaling pathways associated with a particular clinical phenotype.

## Results

### Algorithm overview

**Fig. 1:**
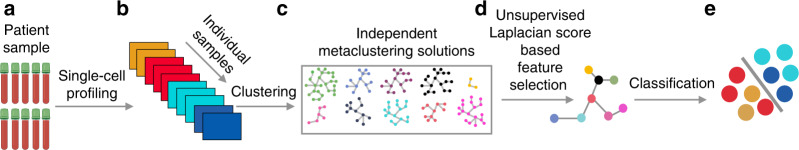
**An overview of the VoPo pipeline for robust clinical outcome prediction**. VoPo is an end-to-end bioinformatics pipeline for prediction and visualization of high-throughput single-cell data. **a** Patient samples are collected and the immune system is profiled at a single-cell level (image created with BioRender.com). **b** Cells from individual samples are first assigned to within-sample clusters. **c** A collection of cell populations (metaclusters) common to all samples is defined through repeated metaclustering. Each uniquely colored graph is a schematic representation of a unique metaclustering solution, with each node representing a metacluster. **d** Unsupervised Laplacian Score-based feature selection is applied independently to each metaclustering solution to reduce feature redundancy. This results in a collection of features acquired collectively across the independent metaclustering solutions. The color of a node (e.g., retained metacluster) schematically represents the metaclustering solution where it was produced (from **c**). **e** The features retained across independent metaclustering solutions are integrated and used to classify patients according to clinical phenotype.

**Fig. 2:**
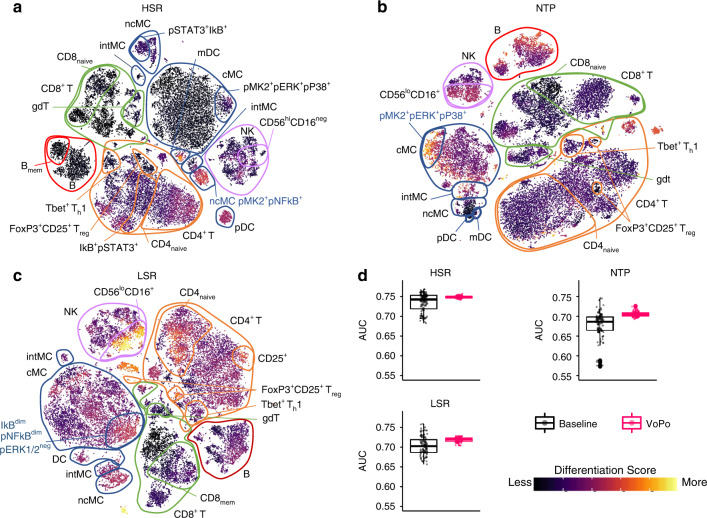
**Applying VoPo to three mass cytometry datasets**. Single-cells across samples were projected into two dimensions using tSNE in the HSR (**a**), NTP (**b**), and LSR (**c**) datasets. Cells were colored by their computed differentiation score, which depicts the degree of association with the clinical outcome of interest. Cells that are brightly colored likely belong to cell populations that are strongly associated with a particular clinical outcome. The direction of frequency differences between case and control samples is shown in Supplementary Figs. 7, 10, and 15. **d** Integrating features extracted over the fifty metaclustering solutions with VoPo (pink boxplots) resulted in higher classification accuracy (AUC) over the baseline case where VoPo was not applied (black boxplots) across the three datasets. The boxplots show median values, interquartile range, whiskers of 1.5 times interquartile range, and all individual points.

Following single-cell profiling of a cohort of subjects (Fig. 1a), VoPo performs individual-sample clustering (Fig. 1b), between-sample repeated metaclustering and feature engineering (Fig. 1c), unsupervised feature selection (Fig. 1d), classification (Fig. 1e), and visualization (Fig. 2a–c). VoPo was tested across three diverse immunological datasets profiling immune response to surgery, normal term pregnancy^16^, and recovery from stroke^17^.

VoPo uses a downsampling-free metaclustering strategy. The metaclustering technique allows for the efficient integration of all cells across all samples. All live cells (DNA^+^CD235^−^CD61^−^cPARP^−^) from each sample are first clustered based on both functional and phenotypical markers (Fig. 1b). Cluster centers (e.g., the mean expression of the markers across all cells within a cluster) identified across samples are then re-clustered to form metaclusters (Fig. 1c). As stochastic clustering algorithms produce different cell-to-metacluster solutions across individual runs, the metaclustering step is run multiple times to produce multiple independent cell-to-metacluster solutions for each sample. For further downstream analysis and interpretation, identified metaclusters are curated by an expert investigator and mapped to known cell types based on median marker expression (Fig. 2a–c).

For each metaclustering result, we engineer a set of frequency-based features encoding the proportion of each sample’s cells assigned to each metacluster. Since both phenotypical and functional markers were used in the metaclustering steps, these frequency-based features provide an efficient joint representation of cellular phenotypes and functions. Running VoPo with *K* total clusters per metaclustering iteration with *I* total metaclustering solutions results in *K* × *I* total engineered frequency-based features. As a result of repeated metaclustering, the engineered features are distinct yet highly correlated. To reduce redundancy, an unsupervised feature selection approach^18^ is applied independently to the set of engineered frequency features produced by each metaclustering solution (Fig. 1d). Metaclusters passing feature selection are used to construct a sample-by-feature matrix to be input to a classification pipeline (Fig 1e). An ensemble leave-group-out cross validation strategy is used to make multiple predictions for each sample (see “Methods” section).

### Applying VoPo to three mass cytometry datasets

VoPo was tested in three datasets measuring whole system peripheral immune responses through mass cytometry^19^. This assay allows for the detailed phenotyping of major innate and adaptive immune cell subsets and assessment of intracellular signaling activities (Supplementary Tables 1, 2, and 3). Mass cytometry has shown to be useful in a number of translational settings, including graft versus host disease^20^, autoimmune diseases^21^, vaccine response^22^, and selective T-cell differentiation^23^. In this work, the tested datasets span diverse clinical applications, including, hip surgery recovery (HSR)^24^, normal term pregnancy (NTP)^16^, and longitudinal stroke recovery (LSR)^17^. For direct comparison, all three datasets were evaluated with a case–control analysis. That is, a supervised binary classification task was formulated based on the frequency-based features computed across metaclustering solutions.

**Fig. 3:**
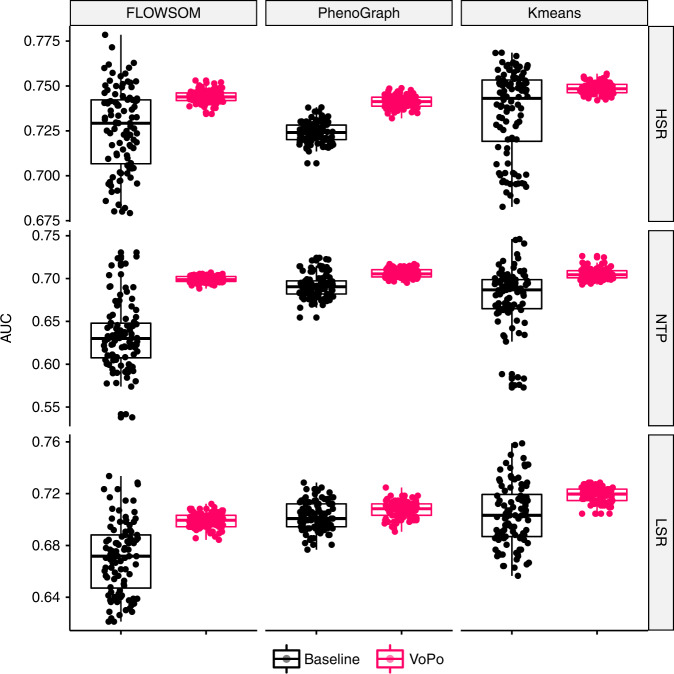
**Applying the VoPo framework across clustering algorithms**. Implementing repeated metaclustering within the VoPo framework improved accuracy and increased robustness across datasets and clustering algorithms. Regardless of whether PhenoGraph, FlowSOM, or *k*-means was used in the clustering and metaclustering components of VoPo, repeated metaclustering with VoPo (pink boxplots) lead to higher average classification accuracy (AUC) with less variability over the baseline case where VoPo was not applied (black boxplots). The boxplots show median values, interquartile range, whiskers of 1.5 times interquartile range, and all individual points.

We studied how integrating features generated through repeated metaclustering leads to higher classification accuracy than the average of those obtained from single metaclustering solutions. Fifty metaclustering solutions were generated with *K*′ = 50 metaclusters in the HSR, NTP, and LSR datasets, respectively. After applying unsupervised locality-preserving feature selection to the collective set of metaclustering-based features to retain 40 clusters per metaclustering iteration (see Supplementary Figs. 3 and 17), the classification pipeline was run and area under the receiver operator curve (AUC) was used as the metric of success. The single clustering (black) and repeated-clustering (pink) distributions of AUCs are visualized for each of the three datasets in Fig 2d. To generate the distribution of baseline classification accuracies (black), we repeatedly selected a random individual metaclustering solution from the fifty that were generated and input its associated features to the classification pipeline. The procedure was repeated 100 times with each datapoint corresponding to a unique, randomly selected metaclustering solution from the fifty that were generated. Similarly, the VoPo classification accuracy distribution (pink) was generated by inputting the frequency features (retained after feature selection) across all 50 metaclustering solutions into the classification pipeline over 100 trials. Across the three datasets, features obtained through repeated metaclustering with VoPo lead to higher classification accuracy over the baseline, with mean AUCs of 0.75, 0.71, and 0.72 in the HSR, NTP, and LSR datasets, respectively. This compares to the mean baseline AUCs in the HSR, NTP, and LSR datasets of 0.74, 0.68, and 0.70. Notably, the variance in classification accuracy was significantly decreased by building models based on features engineered from all metaclustering solutions, as opposed to choosing any of the individual solutions. VoPo also resulted in higher classification accuracy over several baseline algorithms across datasets (Fig. 3, Supplementary Figs. 4–5).

### Visualizing the immune correlates of clinical outcome

To study the immunophenotypes associated with a clinical outcome, VoPo computes user-defined statistics on the identified cell populations and maps this information onto individual cells. Such an approach enables a classic single-cell visualization with additional information related to the clinical variable of interest, as opposed to cluster-level visualization^3–5^. In each dataset, thirty-thousand cells were extracted across samples and projected into two dimensions with t-SNE^25^ based on both phenotypical and functional marker expression (Fig. 2a–c). Cell populations were annotated using phenotypic and functional marker expression (see Supplementary Figs. 6, 9, and 14 for HSR, NTP, and LSR datasets, respectively). While we used t-SNE, the visualization step is compatible with a variety of dimensionality reduction algorithms (Supplementary Figs. 8, 11, and 16). Then, each cell is colored based on its association with the clinical phenotype of interest by computing a differentiation score. First, a similarity measure is computed between each cell and each identified cell-population (metacluster) based on both phenotypic and functional markers. Each cell-population identified through metaclustering has an associated significance score (e.g., fold change) reflecting the extent of frequency difference between case and control samples (see “Methods” section). The differentiation score for a particular cell is a linear combination of the significance scores for each cluster, where the corresponding weights are proportional to the cell-to-metacluster similarity. The resulting visualization allows for the efficient identification of the particular cell types that can differentiate patient phenotypes.

Because clustering is performed using live cells, all major innate and adaptive immune cell populations contribute to the classification task. For the purpose of interpretation, CD45^+^CD66^+^ granulocytes, which are the most frequent among all cells, are excluded during the visualization steps to ensure comprehension and enhance identification of smaller populations.

### Results in the HSR, NTP, and LSR datasets

Longitudinal peripheral blood samples were collected from 58 patients undergoing hip surgery. Samples were collected before and at 1, 6, 24, 48 h, and 2 weeks after surgery. Patients were either given the glucocorticoid (GC) methylprednisolone (*N* = 30) or a placebo treatment (*N* = 28)^24^. This dataset consisted of 115 million cells from 331 samples. The respective classification task was to predict whether a sample was collected from a patient (at any time point) receiving GC or placebo treatment. Visualization of individual cluster frequency differences between the two patient groups (Fig. 2a) revealed broad alterations in innate and adaptive immune cell frequencies. Consistent with prior studies, the most prominent changes included decreased frequencies of monocytes (including classical, cMCs, and nonclassical, ncMCs, monocytes) and CD4^+^ T cell subsets (Supplementary Fig. 7) in GC-treated patients. These cell types contain high levels of the signaling protein inhibitor of nuclear factor-kB (IkB), consistent with known mechanism of action of GCs in innate and adaptive cells^26^.

Ninety-three longitudinal peripheral blood samples from 31 women were collected during the first, second and third trimester of pregnancy. Samples were also collected six weeks post-partum^16^. The analysis classified first trimester (*N* = 31) from the second trimester (*N* = 31) samples. This resulted in 18 million cells over 62 samples included in the analysis. The most prominent differences (Fig. 2b) included a decrease (Supplementary Fig. 10) in CD56^lo^CD16^+^ NK cells, and B cells, and an increase in cMC frequencies in second trimester compared to first trimester samples. These changes between the first and second trimester have previously been reported and are associated with dynamic immunological adaptations to pregnancy^27^. To further connect VoPo’s performance on the pregnancy dataset back to previous work^16^, we also compared third trimester to postpartum pregnancy samples (Supplementary Figs. 12 and 13) and observed similar prioritized cell populations.

Longitudinal peripheral blood samples were collected from 24 patients who suffered from an ischemic stroke^17^. Samples were collected for 1, 2, 3, 7, 14, 90 days, and 1 year after the stroke. In this dataset, the one-year time point serves as a surrogate for the state of a patient’s immune system before a stroke. Samples collected at 48 h (*N* = 22) were classified from one year (*N* = 18) samples. For this comparison, 40 samples from the cohort were included, resulting in 13 million total cells. Consistent with prior analyses of the LSR dataset, the most prominent differences between 48 h and 1 year samples included decreased frequencies (Supplementary Fig. 15) of NK cells and ncMC, and increased cMC and B cell frequencies 48 h after stroke (Fig. 2c). These differences are indicative of an acute inflammatory phase of the stroke immune response 1–5 days after the stroke, characterized by the relative decrease in abundance of regulatory subsets such as NK cells, ncMC, and expansion of proinflammatory cMCs^17^.

In addition to recapitulating findings from prior studies, VoPo revealed differences between patient groups that were overlooked with previously applied gating strategies, and clustering approaches. For example, in the HSR dataset, a subset of activated ncMCs characterized by high pNFkB and pMK2 levels had markedly decreased frequencies between GCs and placebo-treated patient groups, highlighting differential effects of GCs in functionally-distinct monocyte subsets (Fig. 2a). In the NTP dataset, examination of the t-SNE plots colored by differentiation score revealed a subpopulation of cMCs expressing a combination of three functional markers (pMK2, pP38, and pERK1/2) that differed between the first and second trimester of pregnancy, which was not apparent in previous analyses of the same dataset (Fig. 2b). This observation is in line with previously reported increased responsiveness in myeloid cell populations, such as the progressive activation of monocytes throughout pregnancy^28,29^. In the LSR dataset, the VoPo analysis revealed differences in the CD56^lo^CD16^+^ NK cell subpopulation that differed between the two observational time points after stroke and were not identified in a prior analysis (Fig. 2c). This finding is particularly clinically relevant as depletion of systemic NK cells early after stroke due to their mobilization to the ischemic brain and their local inflammatory activity has been associated with post-stroke recovery^30,31^.

Overall, cell populations uniquely identified with VoPo were often defined using a combination of phenotypic and functional markers (e.g. subsets of populations labeled in blue in Fig. 2a–c, e.g., IkB^dim^ pNFkB^dim^ pERK1/2^−^ cMCs in LSR dataset). These findings highlight an advantage of using an unsupervised clustering approach that combines phenotypic and functional information obtained at the single-cell level. Such information enables the identification of immune cell populations with biologically-meaningful differences between clinical study groups. Detailed descriptions of cell populations associated with the particular clinical phenotype and prioritized by VoPo are shown in Supplementary Figs. 19 and 20.

### Computational considerations

One particular advantage of VoPo is its efficient run-time and scalability to a large number of samples. As a result of the metaclustering strategy, the automated cell-population discovery task can be easily parallelized across individual samples for efficient implementation. In the analysis of the HSR, NTP, and LSR datasets with 331, 62, and 40 samples, respectively, the clustering, feature selection, and classification pipelines were parallelized and run on a computer with 35 AMD EPYC 7551 CPUs. The repeated metaclustering process resulted in run-times of 39.63, 7.55, and 5.32 min, respectively. Similarly, performing unsupervised feature selection followed by cross-validated classification resulted in run-times of 115.2, 20.01, and 12.13 s in the HSR, NTP, and LSR datasets, respectively.

Automated cell-population discovery with VoPo lead to features that facilitate superior classification accuracy over several baseline methods (Fig. 3 and Supplementary Figs. 4–5). Importantly, we also observed that particular unsupervised clustering algorithm used is not as important as how it is being applied. In particular, regardless of the clustering algorithm used, integrating features engineered through the VoPo pipeline (pink) lead to higher classification accuracy with less variability over the baseline, where only a single metaclustering solution was used (black). In Fig. 3, we repeated the analysis shown in Fig. 2d. In these experiments, we compared using *k*-means in both individual sample and metaclustering steps to the results obtained using PhenoGraph or FlowSOM clustering. The grid of distributions in Fig. 3 shows classification trials of the baseline distribution of AUCs (black) compared to 100 classification trials from applying repeated metaclustering through VoPo (pink). Across all three datasets and clustering algorithms, implementing repeated metaclustering through the VoPo pipeline across *k*-means, FlowSOM, and PhenoGraph results in higher classification accuracy, on average, compared to the baseline results obtained from using individual metaclustering solutions (black).

**Fig. 4:**
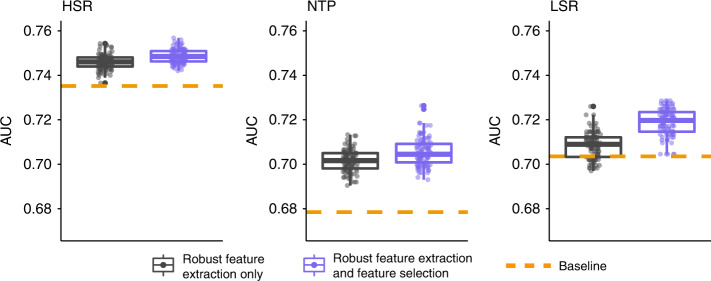
**Applying unsupervised Laplacian score features selection to VoPo features**. Unsupervised feature selection was applied independently to the frequency-based features engineered from each metaclustering solution. Here, we show distributions of classification accuracies (AUC) with (purple boxplots) and without (gray boxplots) feature selection applied to features extracted with VoPo. The dashed orange horizontal line shows the mean baseline classification accuracy using the features obtained without the use of the VoPo pipeline. In all three datasets VoPo’s robust feature extraction (gray) improved the results over baseline (orange boxplots). Additionally, VoPo’s robust feature selection followed by unsupervised feature selection further improved classification accuracy (purple boxplots). The boxplots show median values, interquartile range, whiskers of 1.5 times interquartile range, and all individual points.

We further investigated how applying unsupervised feature selection to the entire collection of features generated through repeated metaclustering can lead to superior classification accuracy. The unsupervised locality preserving feature selection approach^18^ was applied to each metaclustering solution to prioritize a subset of features that can best preserve pairwise distances between samples. Such an approach therefore is effective at removing redundant information (Supplementary Fig. 17) that does not contribute additional information to the understanding of interpatient variability (Supplementary Fig. 1), prior to classification. We studied this across the three datasets by looking at the distribution of classification accuracies from the features obtained over the 50 metaclustering solutions with (purple) and without (black) feature selection. The distributions of AUCs over 100 classification trials are visualized in Fig. 4 and show that in all the three datasets, unsupervised feature selection achieves higher classification accuracy. The orange horizontal line in each plot indicates the mean baseline AUC from the features obtained from a single metaclustering solution. Repeated metaclustering alone increases the performance from the baseline in all three datasets, but feature selection leads to additional competitive advantages. Such an analysis suggests that reducing feature redundancy through a filter-based feature selection is valuable for increasing classification accuracy across clinical datasets.

## Discussion

In summary, VoPo exploits the variability encountered across independent metaclustering solutions to enable clinically-relevant prediction and comprehensive visualization from mass cytometry data. In particular, VoPo uses the diversity of solutions returned through repeated metaclustering to extract additional information from all samples for improved classification accuracy. Such an approach prevents a user from having to choose between one of the variable cell-to-metacluster solutions returned by a stochastic clustering algorithm. Additionally, VoPo’s novel single-cell visualization approach can effectively convey the cell types and signaling events associated with a particular clinical phenotype. Such a visualization strategy therefore facilitates unbiased hypothesis generation by mapping cluster-level statistics to individual cells in a novel way. Here, we applied VoPo to three clinical mass cytometry datasets and showed that it is amenable to any clustering algorithm.

While our examples were focused on binary classification tasks, the feature matrix produced by VoPo provides a flexible input for a range of machine learning tasks, such as, multilabel classification and regression tasks. A user can then further compute a range of statistics on the identified cell populations reflecting how they relate to an external variable of interest. In cases where the external variable is continuous, for example, a user could build a regression model based on the engineered features of the identified cell populations and use model coefficients as the statistic of interest. Further, a promising possible extension of VoPo is to create a so-called “multiview” representation of the immune system, where features are engineered separately for each major cell-type and combined using a multiview machine learning algorithm^32^. Such an approach would enable an understanding of each major cell-type and their overall contribution to the prediction of the desired clinical phenotype. Finally, in its current form, VoPo metaclustering and feature engineering must be re-run if new patient samples are collected. An important area of future work is to more readily accommodate new samples by developing an adaptive clustering and feature engineering strategy, based on an existing VoPo metaclustering result.

VoPo provides a general framework for linking particular cell populations to clinical phenotype and inspires a range of future directions. In regards to the cell-population discovery aspect of VoPo, we could further consider how adding in supervision, such as through specifying “landmark cells” and prior knowledge with ACDC^7^ or by providing human insight into the gating process with DeepCyTOF^8^ or flowDensity^33^ can aid in the interpretability of identified cell populations.

## Methods

### Repeated metaclustering

Automated cell population discovery is achieved through repeated unsupervised clustering. Both functional and phenotypic markers are included in clustering to identify cell populations that are consistent across both phenotype and function. Through repeated clustering, we obtain “multiple views” of the immune system and therefore take advantage of the variability that arises across various runs of a stochastic clustering algorithm. To reduce the computational burden of clustering millions of cells across a large number of samples, we use metaclustering. First, we cluster cells from each of the *S* samples so that cells from sample *i* have been assigned to one of *k*_*i*_ clusters. This results in $K=\mathop{\sum }\nolimits_{i = 1}^{S}{k}_{{{i}}}$ total clusters (or coherent cell-populations). The centers for these *K* clusters are then then clustered into $K^{\prime}$ metaclusters, which are intended to represent the functionally and phenotypically coherent cell-populations in the data. In practice we use *k*-means for all clustering steps, but a user is free to use any clustering method for this task.

After having generated $K^{\prime}$ clusters, we repeat this metaclustering step *I* times. We denote ${K}_{{{m}}}^{\prime}$ as the number of metaclusters defined in iteration in *m*. Moreover, repeating the metaclustering step *I* times results in $P=\sum_{{{m}} = 1}^{I}{K}_{{{m}}}^{\prime}$ total overlapping clusters. We subsequently build features for these *P* clusters, which serve as the input to our classification algorithm.

A user is free to use any stochastic clustering algorithm in the metaclustering steps. In this work, *k*-means clustering was used both in the within-sample clustering and metaclustering steps. Each sample was first coarsely clustered into 1000 clusters (*k*_*i*_ = 1000). We then defined ${K}_{{{m}}}^{\prime}=50$ metaclusters for each clustering iteration in the HSR, NTP, and LSR datasets (Supplementary Fig. 18).

### Markers used for clustering

Analogous to what is typically done in manual gating, existing clustering algorithms for flow and mass cytometry data define cell-populations based only on phenotypic markers^2–5^. This ensures that the computationally-identified cell-populations are capturing cell phenotype, where the expression of various functional markers can be studied further. Similar to the process of manual gating, existing algorithms define frequency and functional marker features for each identified cell population. After clustering, two complementary types of features can then be defined for each sample. A frequency feature for cluster *i* in sample *s* denotes the proportion of each sample’s *s* cells that were assigned to cluster *i*. Alternatively, a functional feature for functional marker *j* in cluster *i* in sample *s* is the mean or median expression functional marker expression of functional marker *j* in cluster *i* in sample *s*. As feature engineering in this manner implements what is done in the manual gating process, it ultimately generates a very large number of features. In particular for each sample there will be *K* + *K* × *F* total features, where *K* is the number of clusters and *F* is the number of functional markers.

In this work, we take a different approach where we define cell-populations with both functional and phenotypic markers to identify particular cell-populations that are both phenotypically similar but also exhibit similar patterns of functional marker expression. The motivation for doing this is that we are then able to define only frequency-based features that capture both phenotype and function. For example, if the expression of functional marker *j* is increased in a CD4^+^ T-cell population in a subset of samples then those samples will also have a higher frequency of CD4^+^ T-cells that have high expression of marker *j*.

### Feature engineering from metaclusters

After having defined *P* total metaclusters, we define frequency and functional marker mean expression features for each cluster across all samples. We let **F** be the *S* × *P* matrix of frequency features, where a particular entry *F*_*ij*_ represents the proportion of sample *i*’s cells that were assigned to metacluster *j*. Similarly, assuming that we have *f* functional markers, we define *f* functional marker matrices reflecting the mean functional marker expression across each of the clusters in each sample. Moreover, we let **X**^*t*^ be the *S* × *P* matrix of functional marker expressions for a particular marker, *t*. An individual entry ${X}_{{{ij}}}^{{{t}}}$ is the mean expression of marker *t* in sample *i* in cluster *j*. We can horizontally concatenate each of the *f* matrices into the full functional *S* × (*P* × *f*) marker feature matrix, X^*^ = [**X**^1^∣**X**^2^∣. . . . ∣**X**^f^].

After having constructed the frequency and functional marker matrices, **F** and X^*^, we can use them individually or in combination as the input to classification tasks. One benefit of including the functional markers in the clustering step is that the frequency features also carry information about the signaling activity in each cluster. As we previously stated, the matrix X^*^ has much higher dimension than **F** and can therefore require more sophisticated machine learning techniques, such as, feature selection or regularization for quality classification results. In Supplementary Fig. 2, we show that using the frequency features encoded in **F** is sufficient for high predictive accuracy across our three example datasets.

### Unsupervised feature selection

Due to the high redundancy in the clusters obtained through repeated metaclustering, the constructed feature matrices, **F** and X^*^ have many collinearities, or sets of features that are highly correlated with each other. As a result, feature selection is a principled approach for reducing the high dimensionality of **F** and X^*^, and for mediating the problems introduced by data colliniearity. We chose to use a locality-preserving feature selection method, developed by He et al.^18^. Intuitively, locality-preserving feature selection methods seek to identify a smaller subset of features that maintain the general distribution of between-sample similarities obtained when all features are used. This objective is enforced through the construction of a *k*-nearest neighbor similarity network between the *S* samples. After having constructed the similarity network, a function of the Graph Laplacian^34^ is used to score each feature for its usefulness in maintaining the patterns of between-sample similarity observed in the original data with all features included. In all three datasets, we selected the top 40 out of the 50 total features per metaclustering iteration, according to their computed Laplacian scores (Supplementary Fig. 3).

This unsupervised feature selection approach is performed on the computed frequency-based data matrix prior to classification. It does not have access to any of the patient phenotypes and therefore does not introduce any bias. This feature selection step is used primarily to eliminate redundancy among features. After applying feature selection, we obtain the input for the supervised analysis, which is performed with leave-group-out cross validation.

### Cross validation

In this work, we focus on binary classification problems. That is, each dataset was analyzed as a case–control study between two clinical classes, with *S* total collected samples. We let **y** be an *S*-length vector, where an individual entry *y*_*i*_ ∈ {0, 1} gives the binary classification label for sample *i*. In the presented work, we use a Random Forest classifier. We note a user is free to use any classification algorithm of their choice. While there are a variety cross validation (CV) strategies, we use a ensemble-based leave-group-out approach. At a high level, this approach repeatedly chooses a unique random subset of patients and their corresponding samples to be used for training, and the remaining to be test samples. Our CV approach uses *B* iterations of bootstrapping to iteratively and independently partition all samples into training and test sets. In a particular CV iteration, we choose a half of all patients to be members of the training set. Using the samples corresponding to the selected training patient samples, we train the Random Forest model. We then predict the labels of the samples corresponding to the unused remaining subset of patients not included in the training set. In particular, the predicted values for these samples reflect the probability of belonging to class “1”. We then store these predicted probabilities that correspond to test set patient samples. After completing *B* = 500 bootstrapped cross validation iterations, we have multiple predicted probabilities of belongs to class “1” for each of the *S* samples, noting that a sample only has a prediction for a particular bootstrapped iteration if it corresponds to a patient that was assigned to the test set. We ultimately predict the probability that a particular sample, *i*, belonging to the “1” class as the median predicted probability over all iterations where *i* was included in the test set. Finally, we define a length-*S* vector, y*, where the entry ${y}_{{\mathrm{i}}}^{* }$ encodes the final predicted probability that sample *i* belongs to class “1”. Using the true response vector, **y**, and the vector of predicted probabilities, y*, we can construct an ROC curve and compute area under the ROC curve (AUROC) as the metric of success.

### Visualization pipeline

We developed a visualization pipeline that allows for interpretation of the cell types and signaling pathways contributing to a particular clinical phenotype of interest. Our general approach is to sample a large subset of all cells (30,000) across all *S* samples and to map significance scores computed for each cluster to the single cells. Because we are using tSNE for dimensionality reduction, we can only visualize a limited number of cells across all samples. We note that a user is free to use any dimensionality reduction method of their choice. Downsampling of cells is only required for the visualization aspect of VoPo but all cells are used to engineer features for predictive modeling. In practice, we compute univariate *p*-values for each cluster that reflect frequency or signaling differences among samples in each class.

After sampling 30,000 total cells (excluding granulocytes) across all sample FCS files, we constructed a cell × marker matrix, **A**, where the functional and phenotypic markers are represented across the columns. We then create a two-dimensional embedding of the cells using tSNE on **A**.

Each cell in the LargeVis representation can then be colored by its expression for each of the phenotypic and functional markers to produce a series of plots (one plot per marker). The major cell types were inferred according to phenotypic marker expression.

The frequency of cells in each cluster across each sample is encoded in the matrix **F**. Here we describe a method for specifying a “differentiation score”, which is intended to be some quantitative measurement reflecting the frequency differences in some cell-population between groups that can be used in a qualitative way to gain intuition about important cell types and signaling pathways. Here, we discuss an example using a two-sided Wilcoxon Rank Sum test to quantify the extent of frequency differences between groups, with the caveat being that associated *p*-values are only being used to order cell-populations and not being interpreted in a classical statistical way. Further, a user is free to compute any statistic of choice to reflect the frequency difference of a particular population between two groups. We use simple univariate *p*-values to improve our understanding of which cell types have significant frequency differences between classes. For each of the *P* clusters the univariate *p*-values are computed using a Wilcoxon Rank Sum test^35^. We let ${{\bf{x}}}_{{{a}}}^{{{p}}}$ be the vector of frequencies features for cluster *p* corresponding to samples in the “0” class. Similarly, we let ${{\bf{x}}}_{{{b}}}^{{{p}}}$ be the vector of frequencies for cluster *p* corresponding to samples in the “1” class. After defining these two vectors, the two-sided Wilcoxon Rank Sum test is used to test the null hypothesis that the means of ${{\bf{x}}}_{{{a}}}^{{{p}}}$ and ${{\bf{x}}}_{{{b}}}^{{{p}}}$ are equal. The more significant the *p*-value, the larger the difference in the frequency feature, *p*, between the two classes (“0” and “1”).

To gain insight into the true statistical significance of all features together, we recommend using a regression model and interpreting the *p*-value of the model fit to samples in the test set as a measure of the collective ability of features to differentiate between distinct clinical outcome classes. A statistically significant model *p*-value implies that some combination of features can be used to effectively partition samples from different classes.

After having computed the univariate *p*-value for each of the *P* metaclusters (or your statistic of choice), we seek to map this information onto our single cell visualization. In other words, points should be colored based on some aggregate representation of the *p*-values for each metacluster and a particular point’s likelihood of belonging to a particular metacluster. We quantify the similarity of a point with each cluster based on a function of the euclidean distance between the cluster’s center and the original point in high dimensions. We let **c**_*j*_ be the length-*m* vector encoding the expression for each of the *m* total functional and phenotypic markers in cluster *j*. We similarly let **x**_i_ be the length-*m* expression profile over the *m* functional and phenotypic markers for cell *i*. Then to define *s*_ji_ or the similarity between cluster *j* and cell *i*, we compute, ${s}_{{{ji}}}=\exp (-\alpha | | {{\bf{c}}}_{{{j}}}-{{\bf{x}}}_{{{i}}}| {| }_{2})$. Here, *α* is a user-defined constant.

In each metaclustering iteration, *m*, we compute *s*_*ji*_ for each of the ${K}_{{{m}}}^{\prime}$ clusters, *j* and each cell, *i*. We then assign cell *i* a differentiation score, defined by a linear combination of univariate *p*-values across clusters, computed as, ${p}_{{{i}}}^{{{m}}}=\frac{{\sum }_{{{j}}}{s}_{{{ji}}}{w}_{{{j}}}}{{\sum }_{{{j}}}{s}_{{ji}}}$. Here, *w*_j_ is the *p*-value computed for the rank sum test for cluster *j*. In general, this quantity is a weighted average over all clusters in a particular metaclustering solution, with higher weight given to the *p*-values corresponding to cluster centers that are more similar to the point of interest. Ultimately, we compute ${p}_{{{i}}}^{{{m}}}$ for each *m* of the *I* total metaclustering solutions and determine the final *p*-value for cluster *i* as the average significance score over the *I* metaclustering solutions. Here we describe details for how differentiation scores were mapped onto cells in each dataset, demonstrating the flexibility of the visualization approach.

While our task in the HSR dataset was to classify patients who had received MP treatment from those who had not, we specifically focused on the difference between control and MP samples 6 h after surgery in the visualization. This implies that significance score (*p*-value) for each metacluster was computed based on only the control and MP samples at the 6 hr timepoint. That is, for a particular metacluster, *p*, we let ${{\bf{x}}}_{a}^{p}$ be the vector of frequencies for *p* corresponding to control samples at the 6 hour (6 h) timepoint. Similarly, we let ${{\bf{x}}}_{b}^{p}$ be the vector of frequencies of metacluster *p* for MP samples at the 6hr timepoint. Then the significance score for cluster *p* is the *p*-value of the Wilcoxon Rank Sum test. Moreover, the visualization in Fig. 2a. can be used to understand the differences in cell population (metacluster) frequencies between control and MP samples at the 6h timepoint. This example demonstrates that the visualization method is capable of taking a subset of sample IDs to compute the significance score of clusters and therefore differentiation scores.

The classification task introduced in the NTP dataset was to classify first from second trimester samples during human pregnancy. This is also the comparison reflected in the visualization in Fig. 2b. That is for metacluster *p* we let ${{\bf{x}}}_{a}^{p}$ be the vector of frequencies of metacluster *p* in samples from the first trimester. Similarly, ${{\bf{x}}}_{b}^{p}$ is the vector of cell frequencies of metacluster *p* among second trimester samples. The significance score for metacluster *p* is therefore the *p*-value of the Wilcoxon Rank Sum Test computed between ${{\bf{x}}}_{a}^{p}$ and ${{\bf{x}}}_{b}^{p}$. These significance scores are then mapped to single cells to define differentiation scores.

In the LSR dataset, we classified patient samples collected 48 h from those collected 1 year after stroke. This is also the comparison reflected in the visualization in Fig. 2c. That is for metacluster *p* we let ${{\bf{x}}}_{a}^{p}$ be the vector of frequencies of metacluster *p* in samples from 48 h after stroke. Similarly, ${{\bf{x}}}_{b}^{p}$ is the vector of cell frequencies in metacluster *p* among samples collected. The significance score for metacluster *p* is therefore the *p*-value of the Wilcoxon Rank Sum Test computed between ${{\bf{x}}}_{a}^{p}$ and ${{\bf{x}}}_{b}^{p}$. These significance scores are then mapped to single cells to define differentiation scores.

### Mass cytometry methods

Mass cytometry is a high-parameter single-cell analysis platform that enables the simultaneous interrogation of multiple signaling pathways in precisely phenotyped cell subsets spanning the entire immune system^36^. This technology provides unprecedented opportunities to describe the human immune system as a network of correlated, cell type-specific attributes, and to investigate the functional relationships between cells within and across hematopoietic lineages^37,38^. Mass cytometry combines inductively coupled plasma and time-of-flight spectrometry with cytometry^19^ and allows for the examination of up to 50 analytes simultaneously at the single cell level using metal-isotope-conjugated antibodies.

### Ex vivo whole-blood immuno-assay

Whole blood was collected from study subjects and processed within 60 min after blood draw. Individual aliquots were processed using a standardized protocol for fixing with proteomic stabilizer (SMART TUBE, Inc., San Carlos, CA) and stored at  −80 °C until further processing.

### Sample barcoding and minimization of batch effects

To minimize the effect of experimental variability on mass cytometry measurements between serially collected samples, samples corresponding to the entire time series collected from one participant were processed, barcoded, pooled, stained, and run simultaneously. To minimize the effect of variability between study participants, samples sets of patients matched for control and treatment (HSR dataset), or randomized for time of sampling (NTP and LSR datasets). Further, the run was completed within consecutive days, while carefully controlling for consistent tuning parameters of the mass cytometry instrument (Helios CyTOF, Fluidigm Inc., South San Francisco, CA).

### Antibody staining and mass cytometry

The mass cytometry antibody panel included surface and intracellular antibodies that are used for phenotyping of immune cell subsets and for the functional characterization of immune cell responses. In Supplementary Tables 1–3 we provide a list of antibodies used in the HSR, NTP, and LSR datasets, respectively. Antibodies were either obtained preconjugated (Fluidigm, Inc.) or were purchased as purified, carrierfree (no BSA, gelatin) versions, which were then conjugated in-house with trivalent metal isotopes utilizing the MaxPAR antibody conjugation kit (Fluidigm, Inc.). After incubation with Fc block (Biolegend), pooled barcoded cells were stained with surface antibodies, then permeabilized with methanol and stained with intracellular antibodies. All antibodies used in the analysis were titrated and validated on samples that were processed identically to the samples used in the respective study. Barcoded and antibody-stained cells were analyzed on the mass cytometer.

### Immune cell feature derivation

The mass cytometry data was normalized using Normalizer v0.1 MATLAB Compiler Runtime (MathWorks)^39^. Files were then debarcoded with a single-cell MATLAB debarcoding tool^40^. Manual gating was performed using CellEngine (https://immuneatlas.org/) (Primity Bio, Fremont, CA). In Supplementary Fig. 21, we show the gating strategy originally used for each of the clinical datasets. The names of cell populations in blue were gated across all three datasets. Conversely, cell populations colored orange and green represent populations that were only identified in the HSR or LSR datasets, respectively (see chart). For the VoPo pipeline, the data from each sample were analyzed using manually gated singlet live leukocytes (DNA^+^cPARP^−^CD235^−^CD61^−^). FACS gating strategies have been linked, in the Supplementary Figure legend, to the corresponding data panels in the manuscript.

## Supplementary information

Supplementary Information

Peer Review File

## Data Availability

All datasets used are publicly available through flowrepository under experiment IDs FR-FCM-ZY3Q, FR-FCM-ZY3R, FR-FCM-Z2AT, FR-FCM-ZYSB for the NTP training, NTP validation, HSR, and LSR datasets, respectively.

## Code availability

VoPo was implemented and tested with R version 3.4.4. Code to run all components of VoPo, as well as to reproduce results is provided at https://github.com/stanleyn/VoPo.
