# Supplementary material for: VoPo leverages cellular heterogeneity for predictive modeling of single-cell data
Source: Nat Commun. 2020 Jul 27;11:3738. doi: 10.1038/s41467-020-17569-8 (PMC7385162; doi:10.1038/s41467-020-17569-8)
Supplement: Supplementary file 1 — Supplementary Information [file 41467_2020_17569_MOESM1_ESM.pdf]

Supplementary Information

VoPo Leverages Cellular Heterogeneity for Predictive Modeling of Single-Cell Data

Stanley, Stelzer *et al.*

June 29, 2020

Supplementary Notes

Inter-patient Variability

The classification tasks across these three datasets are of varying levels of difficulty due to differences in inter-patient variability. To gain an understanding of how easily samples between different phenotypic classes separate, we constructed two-dimensional principal component analysis (PCA) plots (Supplementary Figure 1). To generate the PCA plot for each dataset, repeated metaclustering was performed 50 times to generate 50 corresponding sets of cell-to-population partitions (clusters). The cell frequencies for each of these clusters were the features used to project all samples into two dimensions. Each point is colored by its respective class label across the HSR (pink = treatment, gold = control), NTP (pink = first trimester, gold = second trimester), LSR (pink = 48 hours after surgery, gold = 1 year after surgery). The classification task in the surgery dataset is relatively easy in comparison to the pregnancy dataset where it is more difficult.

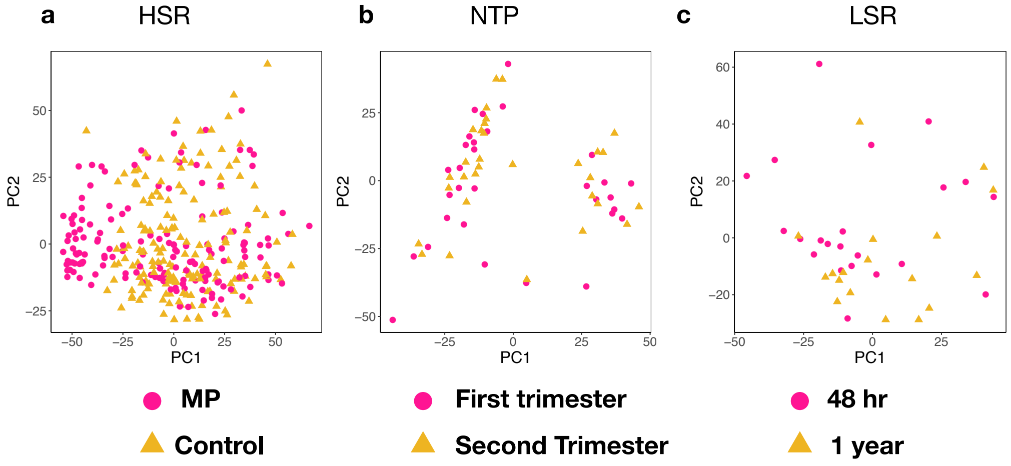

Supplementary Figure 1: **Understanding Inter-Patient Variability Across Clinical Datasets.** PCA was used to project samples into two dimensions using the frequency features constructed across all clusters defined throughout the repeated metaclustering process. The surgery, pregnancy, and stroke datasets are visualized in (**a**), (**b**), and (**c**), respectively. Each point is colored by its respective class with HSR (pink = treatment, gold = control), NTP (pink = first trimester, gold = second trimester), and LSR (pink = 48 hours after surgery, gold = 1 year after surgery).

Feature Engineering for Cell Phenotypes and Cell Signaling Markers

Related work in flow and mass cytometry bioinformatics focuses on constructing two types of features for each cluster. A frequency feature for a particular cluster encodes the number of cells (or the proportion of cells) from each sample assigned to the particular cluster. Each identified cluster is meant to serve as a proxy for a canonical cell-population (i.e. $CD3^+$ T-cells, $CD19^+$ B-cells, etc.). Alternatively, a signaling feature for a cluster represents the mean or median levels of signaling activity in the cluster across samples (i.e. expression of pSTAT5, pERK1/2, etc.). Both types of features are simple to construct from the data and we can therefore consider using these features independently or jointly integrating them (Supplementary Tables 1-3). We refer to frequency features as the set of features that encode cell-to-cluster frequencies in each sample. Alternatively, we refer to joint features as the collective set of frequency and signaling features constructed after clustering cells based on only phenotypic markers. In this case, the set of signaling features for a given functional marker in a particular cluster is the mean functional marker expression in the cluster across samples.

Since we are clustering with both functional and phenotypic markers in VoPo, each cluster represents a particular cell type with distinct signaling behavior. For example, if we have multiple clusters representing $CD4^+$ T-cells, each cluster is distinguishable based on variation in signaling behavior. If only phenotypic markers were used to define clusters, then these subtleties would not be obvious. For this reason, we hypothesized that frequency features by themselves are sufficient for describing the immune landscape across samples. This is indeed the case

and in Supplementary Figure 2 we show that frequency features by themselves lead to high quality clinical outcome predictions, especially in comparison to the joint set of features. We evaluated the performance of frequency and joint features constructed from single iterations of metaclustering. Similar to the experiments described in Figure 2(d) in the main text, we generated 50 metaclustering solutions for each dataset. Then for each feature type we built a distribution of classification accuracies. In each of the 100 trials used to create the classification accuracy distribution, we chose a random iteration of metaclustering and used its associated set of features in the classification task. Across each of the three datasets, the distributions of accuracies (quantified with AUC) are visualized using frequency features (purple) and joint features (gray). Frequency features offer adequate performance and significantly superior performance over the joint set of features in the surgery and pregnancy datasets. This behavior could be influenced by the fact that there are significantly many more joint features than frequency features. Since we use a random forest in our classification task, data that have significantly higher dimensions may lead to higher variability in the process of training the random forest model. As a result, this may lead to lower classification performance. This hypothesis has not been rigorously tested but is of interest in future work.

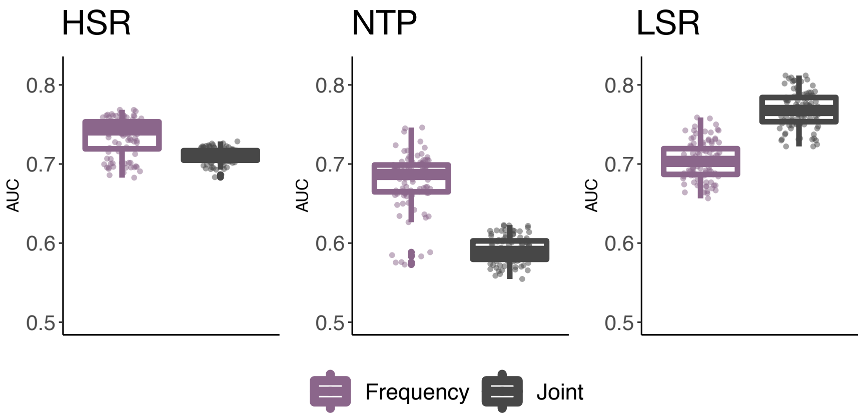

Supplementary Figure 2: **Evaluating Features for Prediction.** The distribution over 100 classification trials using frequency (purple boxplots) and joint frequency/signaling (gray boxplots) features is visualized in all three datasets. In each classification trial, a single metaclustering iteration over all 50 that were run is chosen and the frequency features (purple) or joint features (gray) were used in the classification task. Frequency features offer superior classification accuracy over joint features in the surgery and pregnancy datasets. Classification accuracy with joint and frequency features is similar in the stroke dataset. The boxplots show median values, interquartile range, whiskers of 1.5 times interquartile range, and all individual points.

We also investigated how the number of selected features influences prediction accuracy (Supplementary Figure 3). The number of selected features used in Figure 2d of the main text as well were 10, 10 and 40, respectively. Distributions of classification accuracies were constructed by running the classification pipeline 50 times. The pink horizontal line in each plot shows the mean AUC obtained after integrating all features over 50 independent metaclustering solutions without applying feature selection. The results suggest that across datasets there are multiple appropriate values for the number of selected features that lead to higher classification accuracy over the baseline. Future work could examine what type of feature selection works best for this task and the characteristics of particular datasets that cause them to benefit from more or less selected features.

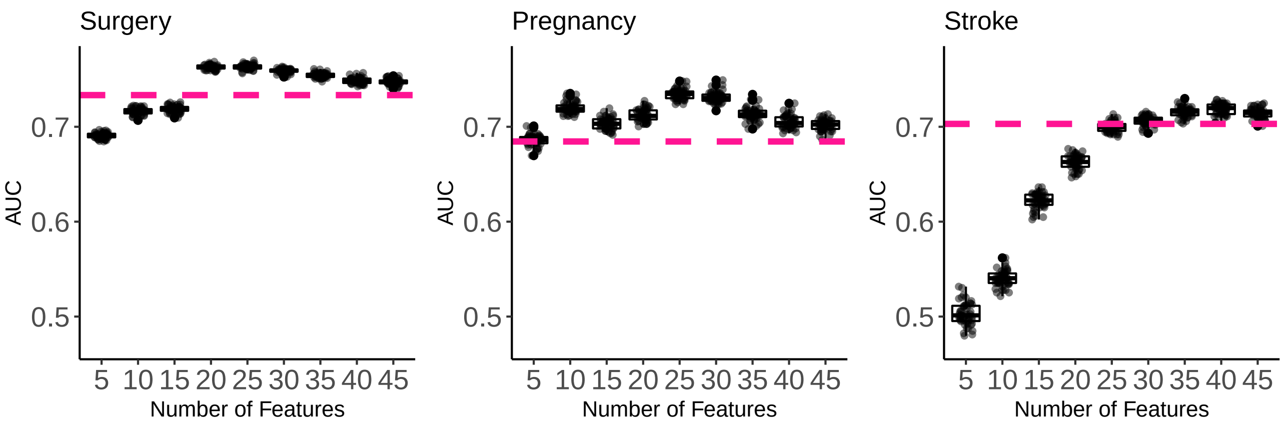

Supplementary Figure 3: **The Effect of the Number of Selected Features on Classification Accuracy.** In each dataset, we tested the effect of the number of features selected and integrated across the independent metaclustering solutions. The dashed pink horizontal line indicates the mean AUC after classification based on the features obtained across all metaclustering iterations without applying feature selection. In all three datasets there are multiple values for the number of selected features that could be chosen to increase the prediction accuracy over the baseline case where no feature selection is used. The boxplots show median values, interquartile range, whiskers of 1.5 times interquartile range, and all individual points.

Comparison with State-Of-The-Art Mass Cytometry Bioinformatics Tools

Current bioinformatics methods for the analysis of mass cytometry data such as, Citrus [1], Spade [2], CytoDX [3], FlowSOM [4], and PhenoGraph [5] are focused on defining coherent cell populations in single-cell data but are not typically applied in clinical classification tasks. In other words, the frequency or signaling-based features defined by these clustering algorithms were not intended to be used in classification tasks. Citrus [1] is the exception, where the authors used hierarchical clustering regularized Lasso regression [6] to predict phenotypic characteristics of samples from the constructed clusters. However, Citrus heavily downsamples in order to accommodate the hierarchical clustering step. Alternatively, CytoDX [3] is a recently developed algorithm that also uses regularized regression to predict sample phenotype or clinical outcome based on individual cells. Our method is therefore one of the first that simultaneously uses all cells and constructs features that are well-suited for sample phenotype or clinical outcome prediction.

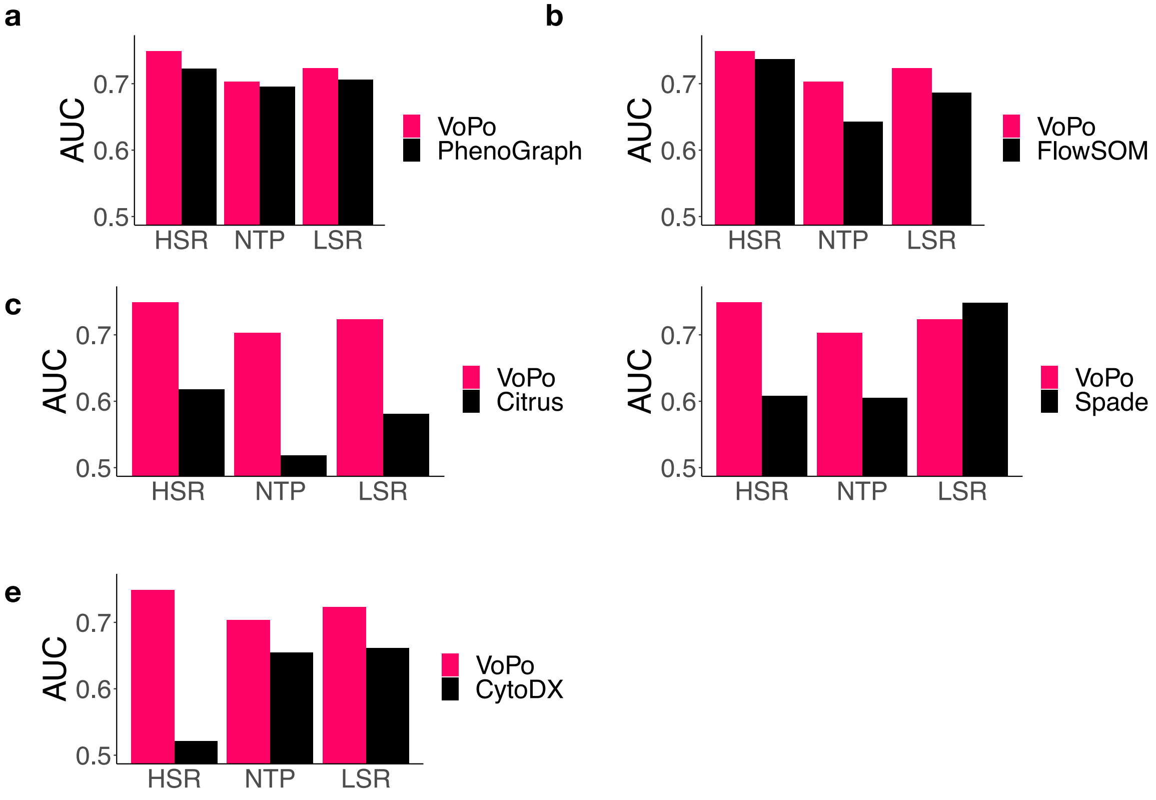

Supplementary Figure 4: **Comparison to State-Of-The-Art Methods.** Repeated metaclustering leads to higher classification accuracy compared to state-of-the-art single-cell clustering algorithms (**a.**) PhenoGraph, (**b.**) FlowSOM, (**c.**) Citrus, (**d.**) Spade, and (**e.**) CytoDX and across all three clinical datasets. In each panel, pink bars correspond to VoPo performance and black bars correspond to the performance of the indicated algorithm being compared.

To study how repeated metaclustering compares to the standard algorithms in the field, we compared its classification accuracy to that of Citrus [1], Spade [2], CytoDX [3], FlowSOM [4], and PhenoGraph [5] by constructing ROC curves. For VoPo, Citrus, Spade, PhenoGraph, and FlowSOM, the cross validation pipeline was used to generate a vector of prediction probabilities for each sample. This concept is described in more detail in the Methods section. The prediction probability for a sample reflects the probability that it belongs to the '1' class according to the model. A distribution of classification accuracies was created by running the cross validation pipeline 25 times using the features generated by each clustering algorithm. This produced 25 different prediction probability vectors for each algorithm. To generate ROC curves, we took the predicted probability for each sample to be the median predicted probability over the 25 runs of the cross validation pipeline. Since CytoDX is not based on clustering and operates on a single cell level, we adopted a similar approach and ran the CytoDX pipeline with our cross validation strategy 25 times to generate 25 prediction values for each sample and ultimately used the

median predictions across samples to construct ROC curves.

Area under the ROC curve (AUC) was used to quantify the classification accuracy of repeated metaclustering compared to PhenoGraph, FlowSOM, Citrus, Spade, and CytoDX in each of the three datasets (Supplementary Figure 4**a-e**). Repeated metaclustering with VoPo offers superior performance across the majority of datasets and algorithms. This was further jointly visualized by plotting the ROC curves of all methods in the HSR, NTP, and LSR datasets (Supplementary Figure 5**a**) and the corresponding AUC scores (Supplementary Figure 5**b**).

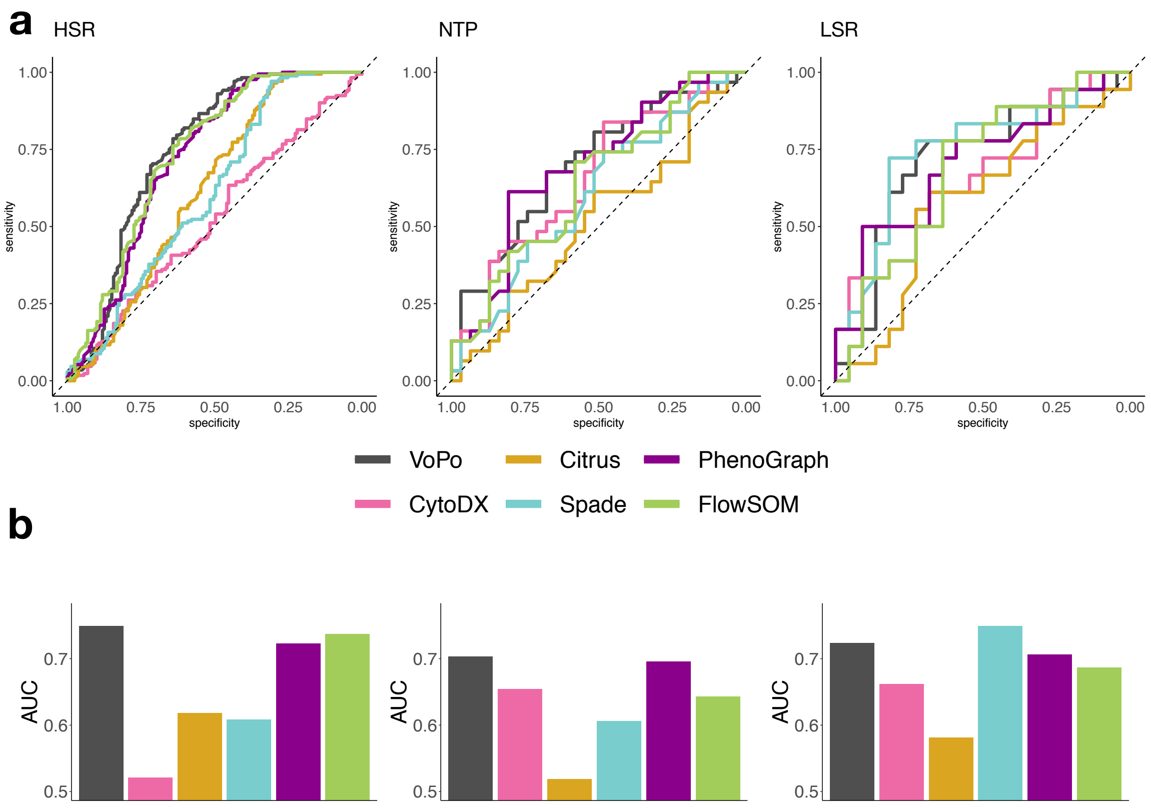

Supplementary Figure 5: **Visualizing ROC curves Across Algorithms and Datasets. a.** The ROC curves were plotted for VoPo (gray) in comparison to several baseline methods (gold, purple, pink, turquoise, and green for Citrus, PhenoGraph, CytoDX, Spade and FlowSOM, respectively. **b.** The corresponding AUCs to the ROC curves shown in (**a**) are plotted per dataset.

VoPo's Novel Single-Cell Visualization

In Figure 2a-c (main text) single cells were projected into two dimensions using tSNE [7]. Cells were then colored by their computed differentiation scores, according to the VoPo clustering result. To interpret our results and to understand how other dimensionality reduction methods could be used, we did the following:

- Plot the expression of all phenotypic and functional markers in each of the cells on the 2-d tSNE plots in Figure 2 (main text)
- Show that we can also map other types of statistics such as directional difference onto the plot
- Show LargeVis as an alternative visualization strategy

Annotating tSNE Visualizations

To annotate the cell types shown in Figure 2a-c (main text), we colored cells in each of the three 2-dimensional visualizations by the expression of each phenotypic and functional marker. Doing so allowed us to annotate known cell-types in the plots. These results can be shown for the HSR, NTP, and LSR datasets in Supplementary Figures 6, 9, and 14.

Mapping Alternative Statistics onto the 2-D Single-Cell Visualizations

In Figure 2a-c (main text), we showed annotated single cell plots for all three datasets that reflect the extent of difference for each cell population between clinical outcome classes. We complemented this analysis by considering the directional differences of the identified cell population (clusters) between clinical outcome classes. That is, we sought to explicitly visualize which clinical phenotype had a higher frequency of a particular cluster. Instead of computing a Wilcoxon p-value to quantify the extent of frequency differences between clinical phenotypes, we computed a *directional score*. We let $\mathbf{x}_a^p$ and $\mathbf{x}_b^p$ be the cell-to-metacluster frequencies for metacluster p in samples from classes a and b, respectively. We compute the directional score S_{dir} as, $S_{\text{dir}} = \log_2(\mathbf{x}_a^p/\mathbf{x}_b^p)$.

In Supplementary Figures 7, 10, and 15, we show the directional difference plots (left) along with the original differentiation score plots (right) visualized in Figure 2d (main text). Individual cells were mapped across all identified clusters in a manner analogous to what was done in the plots where cells were colored according to differentiation scores. For a particular metacluster, p, we computed its directional difference score as S_{dir}. The resulting value for each cell used for its coloring was therefore computed as a linear combination of the directional differences computed for each cell population (cluster), where the weights in the linear combination reflected the pairwise similarity between cluster and cell. The ultimate value of a cell was then the average of this process over independent clustering solutions with purple and gold used communicate the clinical phenotype with higher frequency. We have shown points colored according to cluster p-values or directional difference but a user is free to incorporate any other statistic into the visualization. A possible suggestion is a differential abundance score similar to that used in Cydar [8].

VoPo Accommodates a Variety of Dimensionality Reduction Approaches

While we chose to use tSNE as our dimensionality reduction method in Figure 2, a user is free to use the algorithm of their choice to project cells into two dimensions. For each of the three datasets, we also used the LargeVis algorithm [9] to project 30,000 cells into two dimensions. This emphasizes the flexibility of our approach to a variety of dimensionality reduction methods. The LargeVis visualizations for the HSR, LSR and NTP are visualized in Supplementary Figures 8, 11, and 16, respectively.

Visualization of the HSR Dataset

In Supplementary Figure 6 we first show the phenotypical and functional marker expression of single cells used to annotate the HSR plot in Figure 2 (main text).

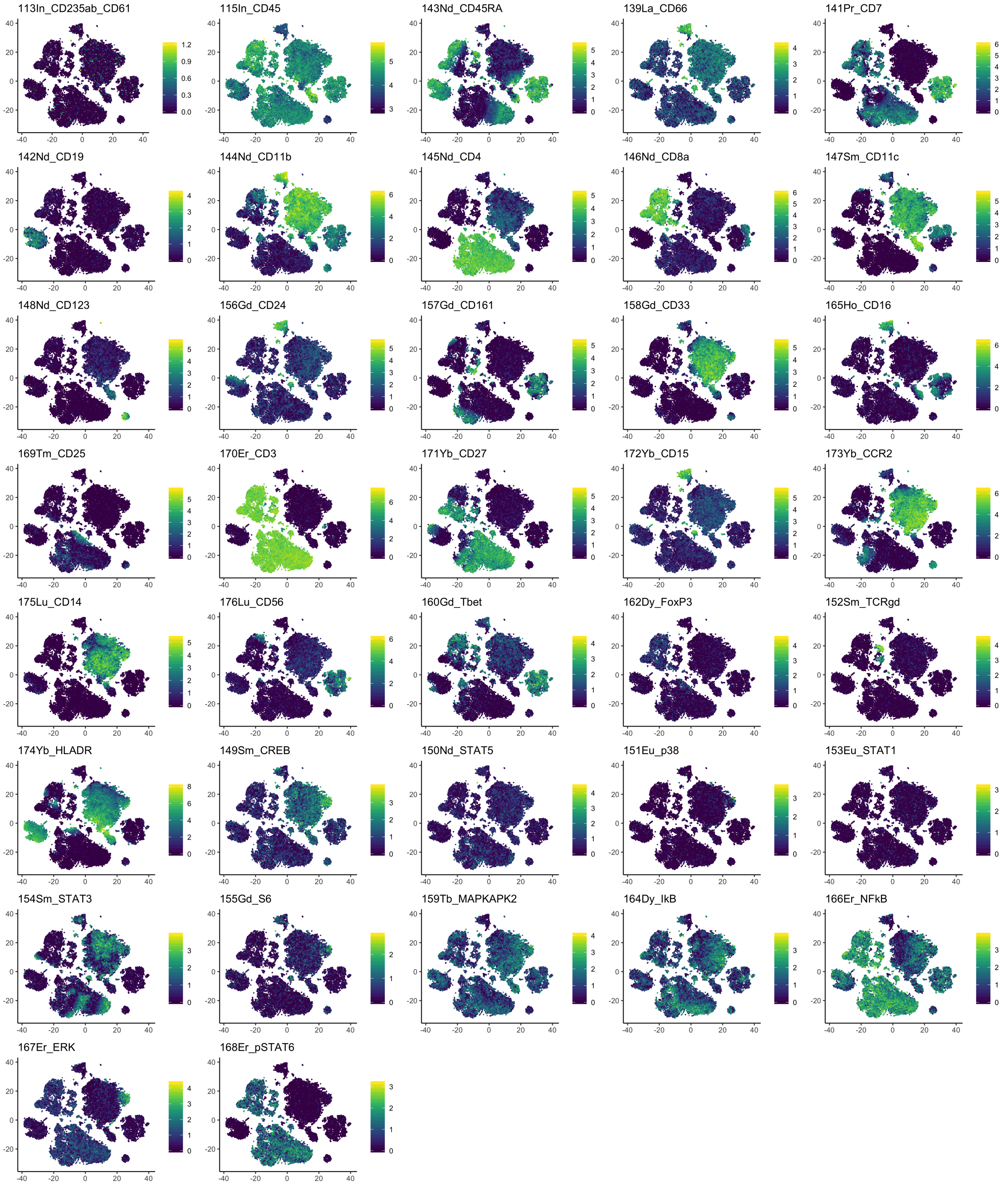

Supplementary Figure 6: **Marker Expression in the HSR Dataset.** Single cells sampled across samples in the HSR dataset and colored by the expression of all functional and phenotypic markers. The color scale represents marker expression.

The directional difference for particular cell types in the HSR dataset were then visualized in Supplementary Figure 7.

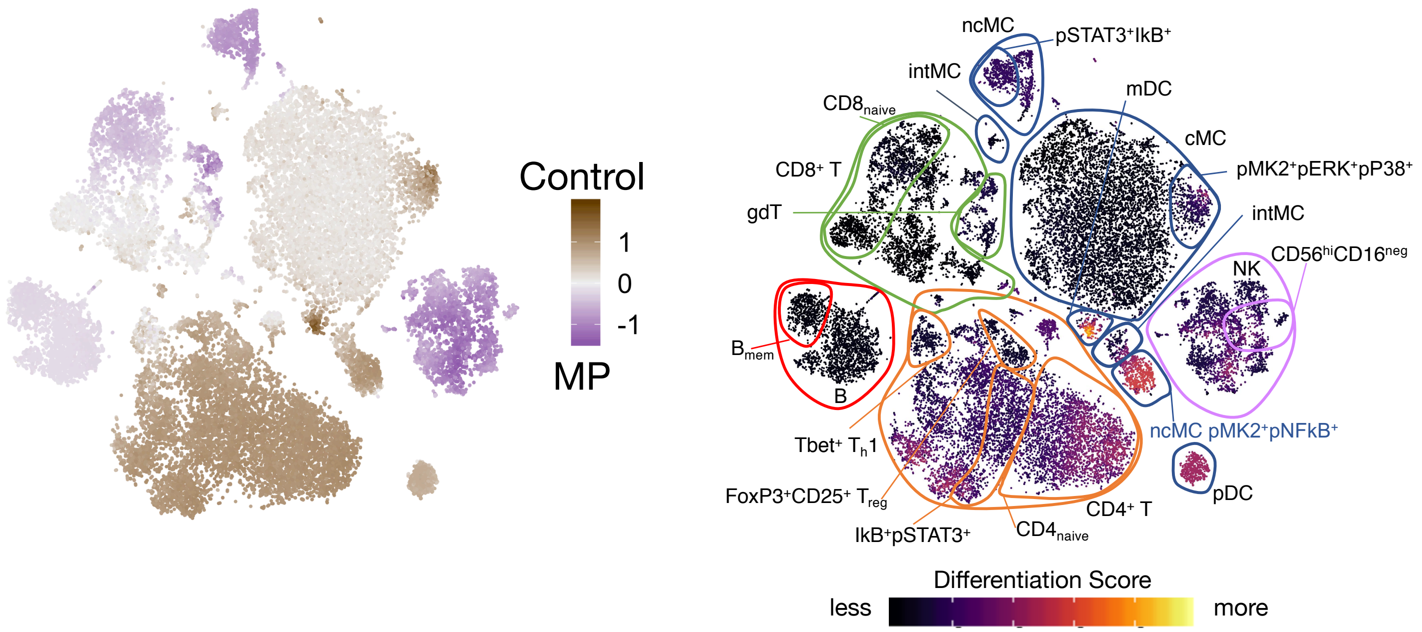

Supplementary Figure 7: **Visualizing Differences Between Clinical Outcome Classes in the HSR Dataset.** Single cells were sampled across samples in the HSR dataset and colored by a mapped directional difference score (left) or a mapped differentiation (right). In the directional difference plots (left), gold represents a higher frequency in control samples, while white indicates equality between control and MP samples. In the differentiation score plots (right), yellow represents higher difference between clinical outcome classes while black symbolizes a smaller difference between clinical outcome classes.

Alternative LargeVis visualizations are then shown for the HSR dataset in Supplementary Figure 8.

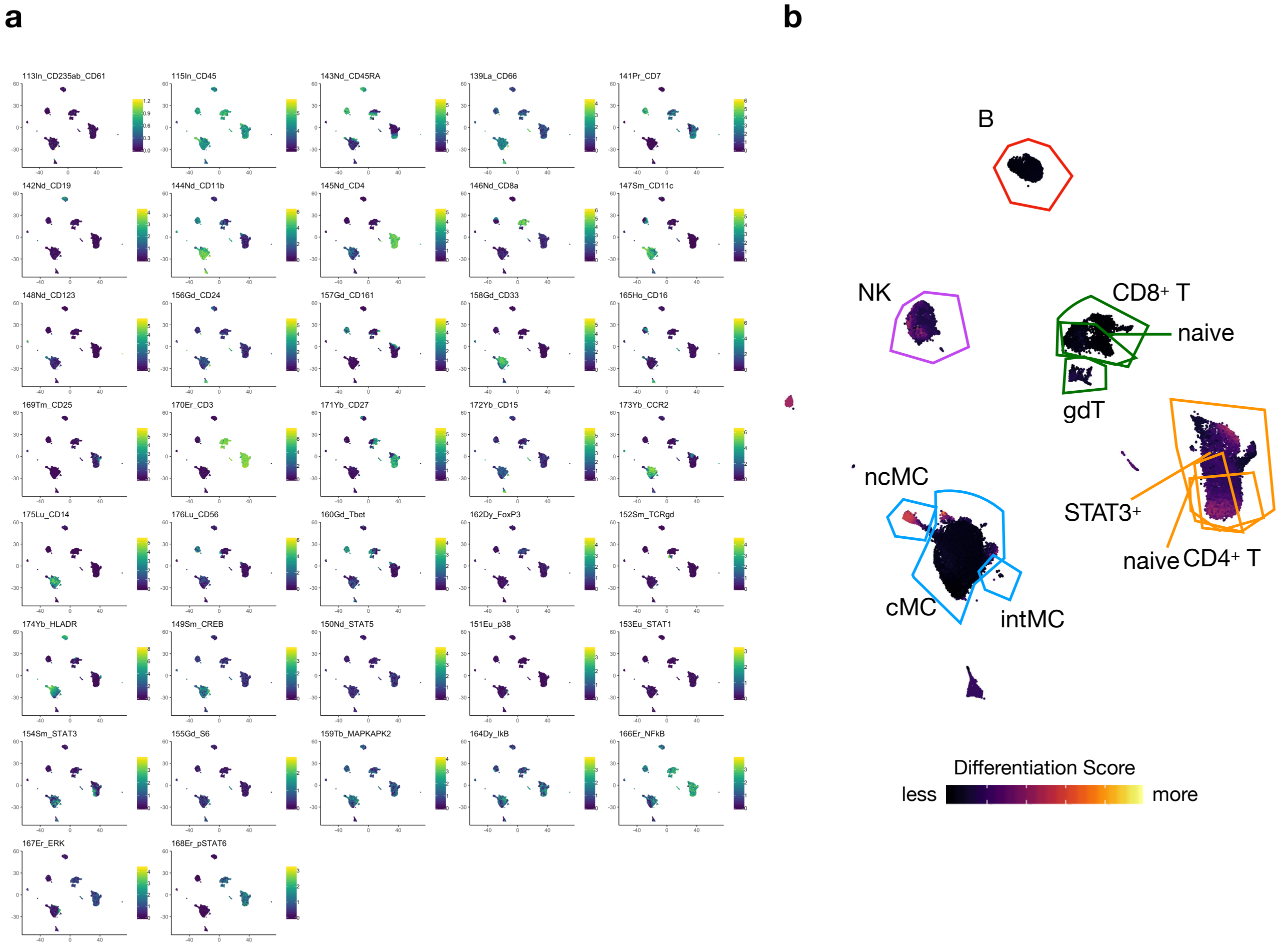

Supplementary Figure 8: **LargeVis Visualization of Single Cells in the HSR Dataset.** **A.** Single cells are visualized in the HSR dataset in two dimensions using LargeVis. Across sub-plots, cells are colored by the expression of each of the phenotypical and functional markers. The color scale represents marker expression. **B.** Cells are colored by their differentiation score.

Visualization of the NTP Dataset

In Supplementary Figure 9 we first show the phenotypical and functional marker expression of single-cells used to annotate the NTP plot in Figure 2 (main text).

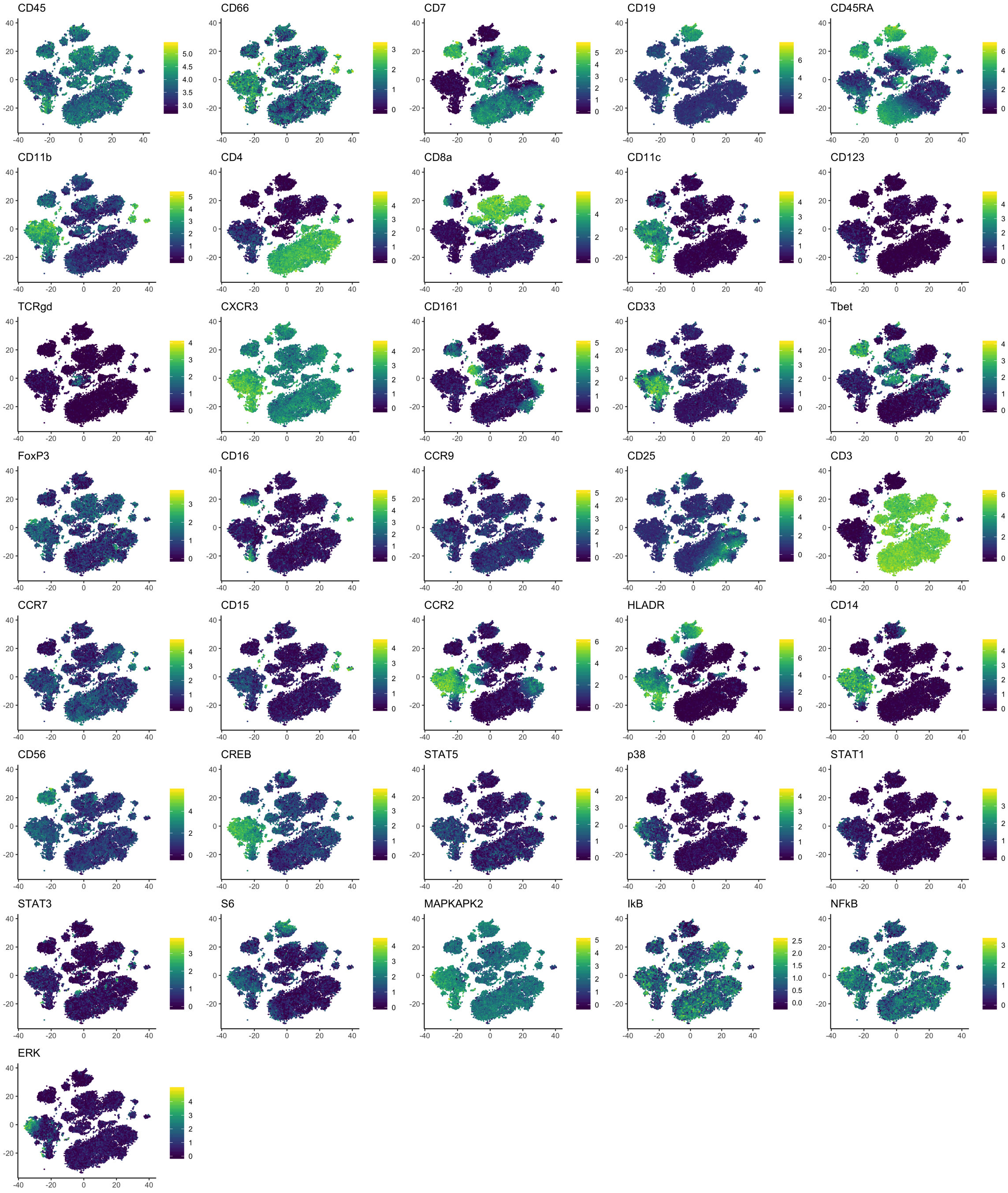

Supplementary Figure 9: **Marker Expression in the NTP Dataset.** Single cells sampled across samples in the NTP dataset and colored by the expression of all functional and phenotypic markers. The color scale represents marker expression.

The directional difference for particular cell types in the NTP dataset were then visualized in Supplementary Figure 10.

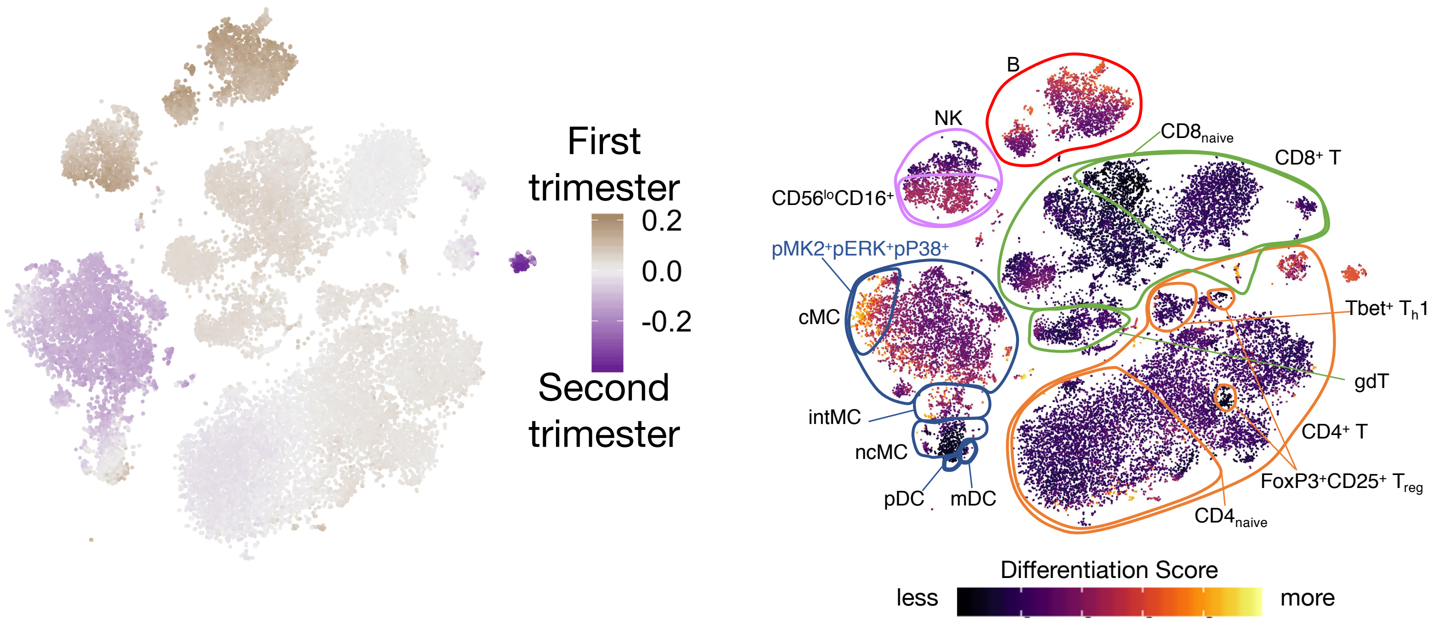

Supplementary Figure 10: **Visualizing Differences Between Clinical Outcome Classes in the NTP dataset.** Single cells were sampled across samples in the NTP dataset and colored by a mapped directional difference score (left) or a mapped differentiation score (right). In the directional difference plots (left), gold represents a higher frequency in first trimester samples while purple denotes higher frequency in second trimester samples. In the differentiation score plots (right), yellow represents higher difference between clinical outcome classes while black symbolizes a smaller difference between clinical outcome classes.

Alternative LargeVis visualizations are then shown for the NTP dataset in Supplementary Figure 11.

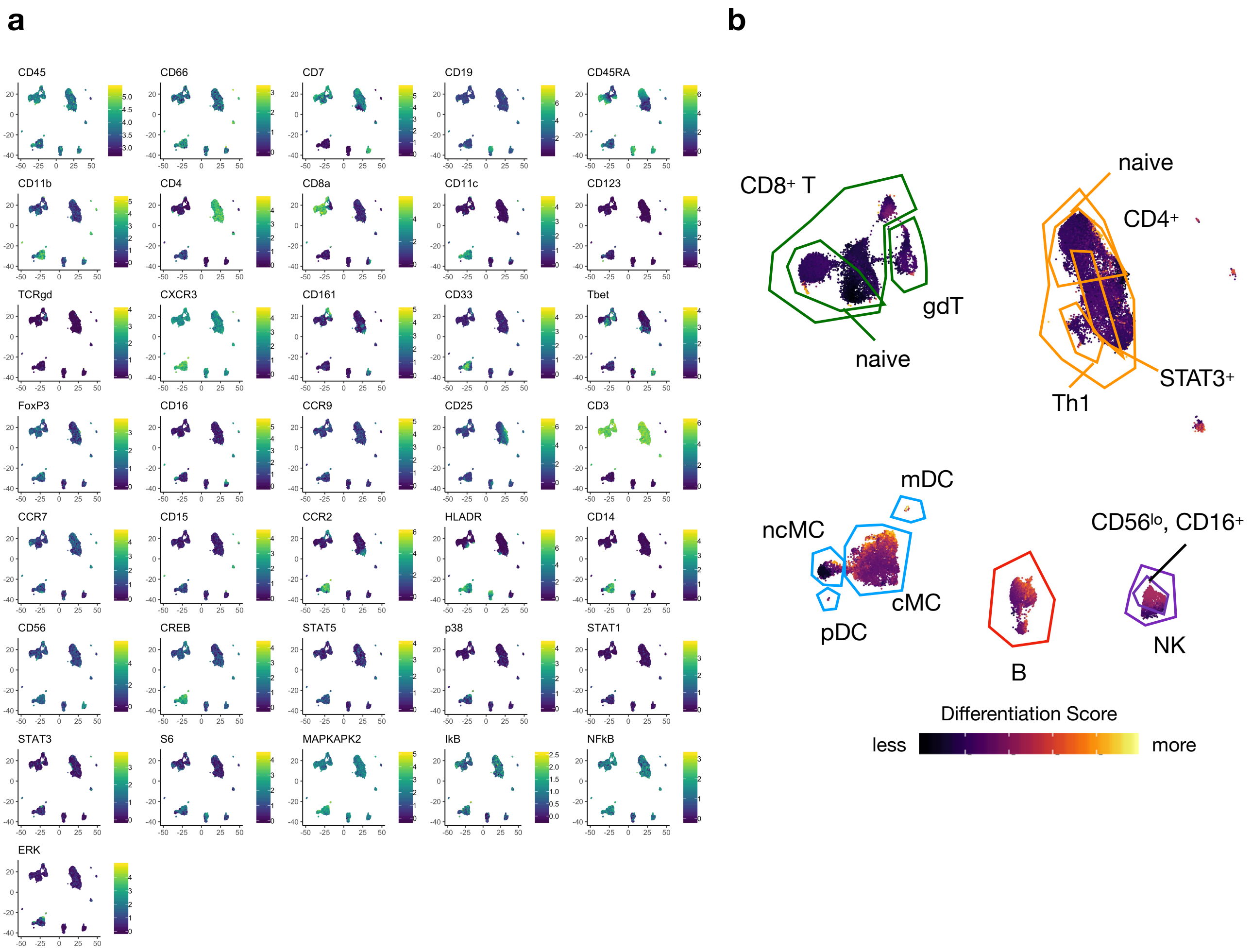

Supplementary Figure 11: **LargeVis Visualization of Single Cells in the NTP Dataset. A.** Single cells are visualized in the NTP dataset in two dimensions using LargeVis. Across sub-plots, cells are colored by the expression of each of the phenotypical and functional markers. The color scale represents marker expression. **B.** Cells are colored by their differentiation score.

Comparing Third Trimester from Postpartum Samples

In the main text, we chose to focus on classifying first from second trimester samples to see how VoPo would perform in such a challenging classification task. To connect VoPo's performance on the pregnancy dataset back to previous work [10] we chose to compare third trimester to postpartum pregnancy samples (Supplementary Figure 12). The analysis classified third trimester ($N = 31$) from samples collected postpartum and revealed differences in cell subset distributions. The most prominent differences included a postpartum increase in NK cells, non-classical monocytes, plasmacytoid dendritic cells (pDCs), and gamma delta T cells ($\gamma\delta$T) frequencies in comparison to third trimester samples. Increases in CD56^{lo}CD16^{+} NK, as well as $\gamma\delta$ T cell populations after delivery have previously been reported [11, 12] and may reflect the maternal immune system's return to non-pregnant conditions as these populations expand over the course of pregnancy.

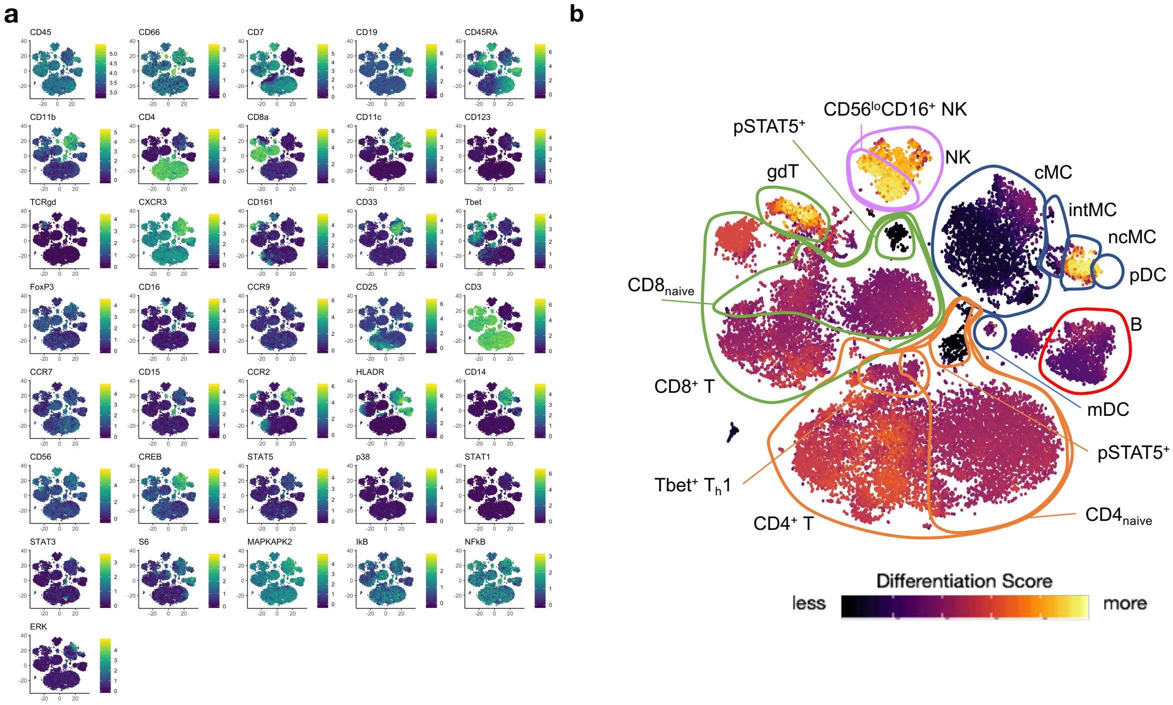

Supplementary Figure 12: **Visualizing Cell-Population Differences Between Third and Postpartum Samples. a.** 30,000 cells were sampled across the collection of third trimester and postpartum samples. Cell-populations were annotated using surface and functional marker expression.The color scale represents marker expression. **b.** VoPo's visualization pipeline was used to understand the cell populations differing between third trimester and postpartum samples. Strong differences were observed in NK cells, non-classical monocytes, plasmacytoid dendritic cells and $\gamma\delta$ T-cells.

We repeated the experiment described in Figure 2d. in the main text on the third trimester versus postpartum comparison. That is, fifty metaclustering solutions were generated and we compared the classification among randomly selected single solutions from the fifty generated to the accuracy obtained from inputting the engineered frequency features into the cross validation pipeline 100 times. Combining features (with feature selection) across all metaclustering solutions resulted in a higher average AUC than the average obtained from single metaclustering solutions (Supplementary Figure 13).

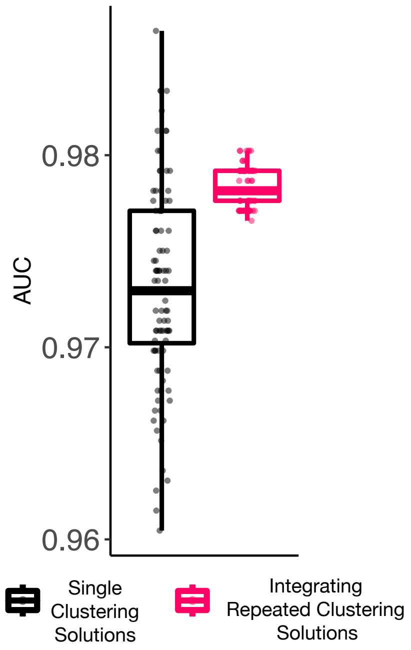

Supplementary Figure 13: **Classifying Third Trimester from Post-Partum Samples.** Integrating repeated metaclustering solutions results in higher mean AUC (pink boxplots) than that obtained using individual clustering solutions (black boxplots) in the third trimester versus postpartum classification task. The boxplots show median values, interquartile range, whiskers of 1.5 times interquartile range, and all individual points.

Visualization of the LSR Dataset

In Supplementary Figure14 we first show the phenotypical and functional expression of single-cells used to annotate the LSR plot in Figure 2 (main text).

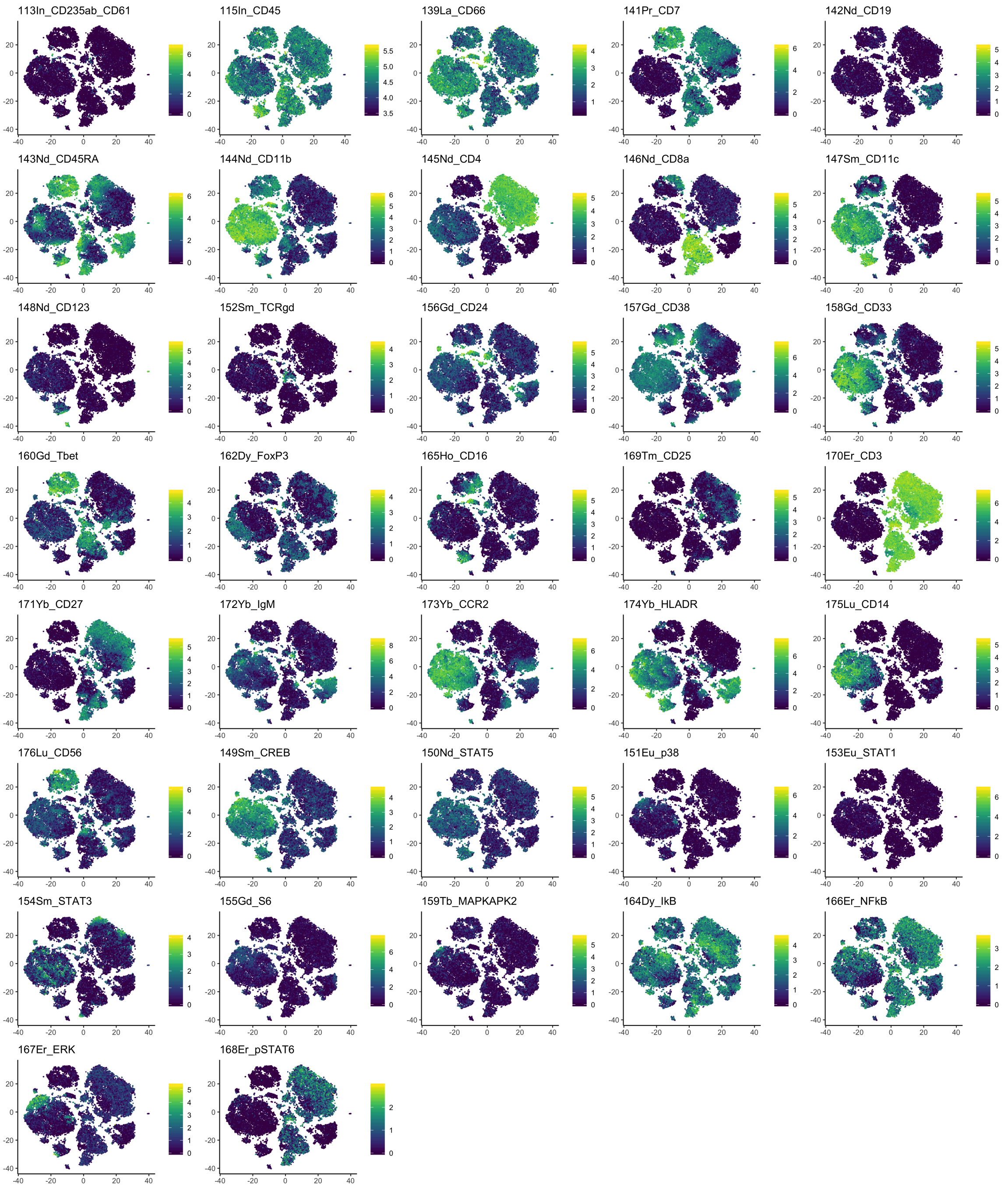

Supplementary Figure 14: **Marker Expression in LSR Dataset.** Single cells sampled across samples in the LSR and colored by the expression of all functional and phenotypic markers. The color scale represents marker expression.

The directional difference for particular cell types for the LSR dataset were then visualized in Supplementary Figure 15.

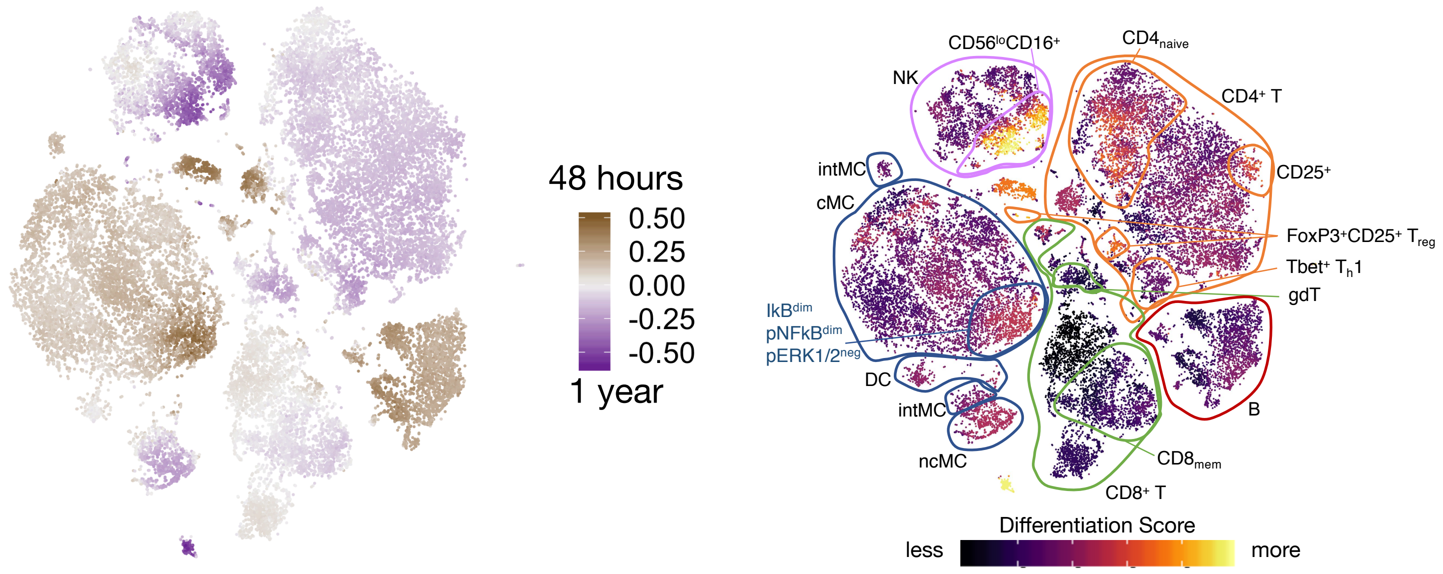

Supplementary Figure 15: **Visualizing Differences Between Clinical Outcome Classes in the LSR Dataset.** Single cells were sampled across samples in the LSR dataset and colored by a mapped directional difference score (left) or a mapped differentiation score (right). In the directional difference plots (left), gold represents a higher frequency in the 48 hr samples while purple denotes higher frequency in the 1 year samples. In the differentiation score plots (right), yellow represents higher difference between clinical outcome classes while black symbolizes a smaller difference between clinical outcome classes.

Alternative LargeVis visualizations are then shown for the LSR dataset in Supplementary Figure 16.

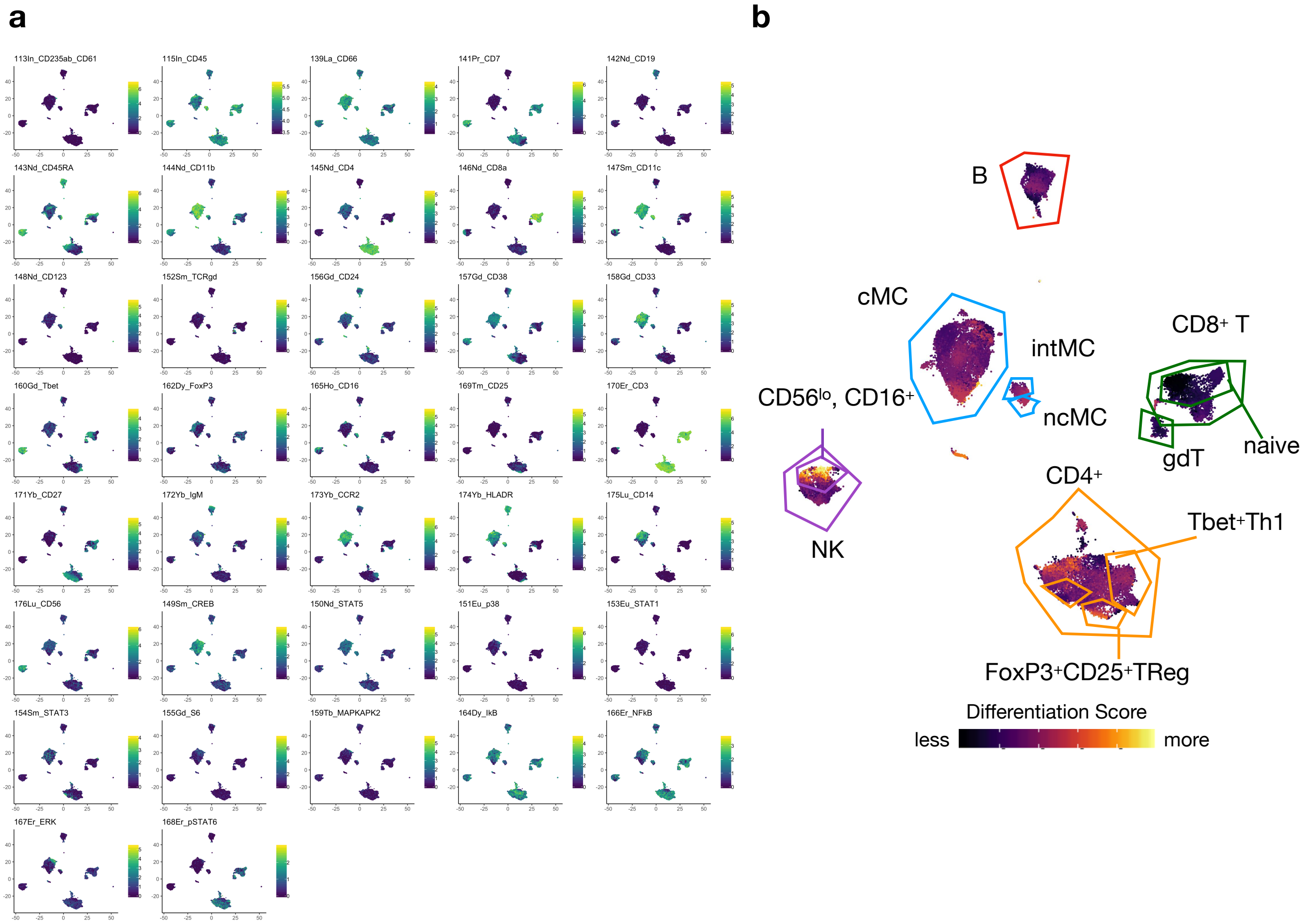

Supplementary Figure 16: **LargeVis Visualization of Single Cells in the LSR Dataset. A.** Single cells are visualized in the LSR dataset in two dimensions using LargeVis. Across sub-plots, cells are colored by the expression of each of the phenotypical and functional markers. The color scale represents marker expression. **B.** Cells are colored by their differentiation score.

Feature Selection Optimization

In Figure 4 (main text) we showed that feature selection aids in classification performance along with repeated metaclustering. In all three datasets, we selected 40 of the frequency-based features out of the 50 features (or 80% of features) that were generated per metaclustering solution. We sought to understand which cell-types were being retained in our unsupervised feature selection strategy. We used the probabilistic mapping strategy (Methods, section 2.7) used to visualize the datasets in Figures 2 **a-c** to communicate the likelihood that each individual cell belonged to a population that was retained through feature selection. To do this, we still matched each cell probabilistically to every metacluster. However, instead of inputting a statistic reflecting the difference between patient phenotype classes, we simply associate a binary indicator for each metacluster, reflecting whether or not it was retained in the feature selection process. Therefore, when we propagate this information onto single cells, we get an idea of which cell-types are ultimately being input into the random forest model for training. The results in Supplementary Figure 17 color each point (cell) according to the likelihood that the cell belongs to a metacluster that was selected by the unsupervised feature section approach. Green points correspond to those cells that likely belong to a metacluster that did not pass feature selection. Alternatively, pink points represent cells corresponding to metaclusters that did pass feature selection.

We found that our unsupervised locality preserving Laplacian-based feature selection approach naturally retains several of the useful features discussed in Figure 2 across datasets. In the HSR dataset, for example, we see prominent inclusion of pDCs, $CD4^{+}$ T-cells, and non-classical monocytes (ncMC). In the NTP dataset, this result showed that NK cells, cMCs and $CD8^{+}$ T-cells were retained. Finally in the LSR datasets, we saw prominent inclusion of NK cells, B cells, ncMCs, for example. Our results suggest that the feature selection approach helps to preserve information across all parts of the immune system.

VoPo's feature engineering and feature selection process has parallels with transductive learning [13, 14]. Transductive learning approaches makes use of of unlabeled test data during the training process to optimize a predictive model, such that samples that are similar in feature space tend to have similar labels [14]. Our joint feature engineering and feature selection approach that is performed prior to the learning phase is reminiscent of transductive learning because it combines all samples to define clusters and corresponding features that are potentially higher quality than that which could be attained by only clustering the cells from the training samples. As a future extension of VoPo, we propose to define the collection of metaclusters on only training instances and to use the extracted metacluster centers to seed cluster centers for test set samples. This so called 'adaptive' clustering approach will not require re-clustering all samples if a new sample was collected. We further see the opportunity to combine this adaptive clustering with a transductive learning approach, similar to Supplementary Ref. [13].

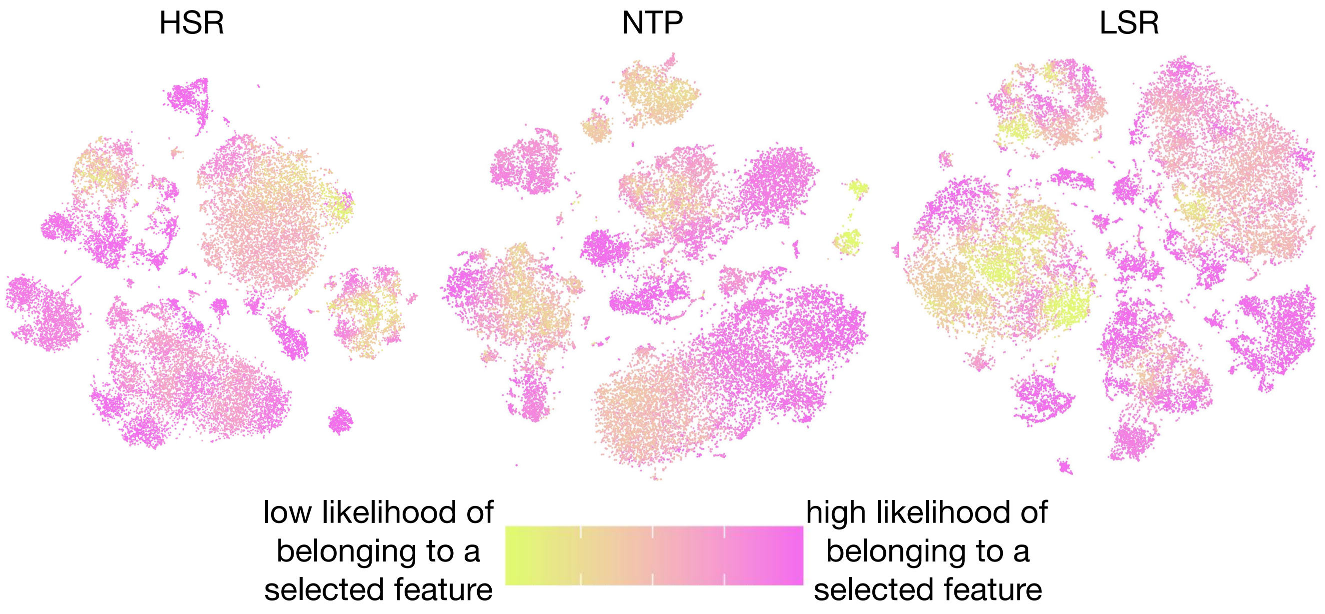

Supplementary Figure 17: **Analysis of Features Retained After Feature Selection.** The probabilistic mapping strategy was used to understand the cell-populations retained after unsupervised feature selection. Cells colored pink are predicted to belong to cell-populations that were likely retained after unsupervised feature selection. Alternatively, green-colored cells belong to populations that did not pass the feature selection process. This analysis shows that the feature selection process is agnostic, and represents all aspects of the immune system.

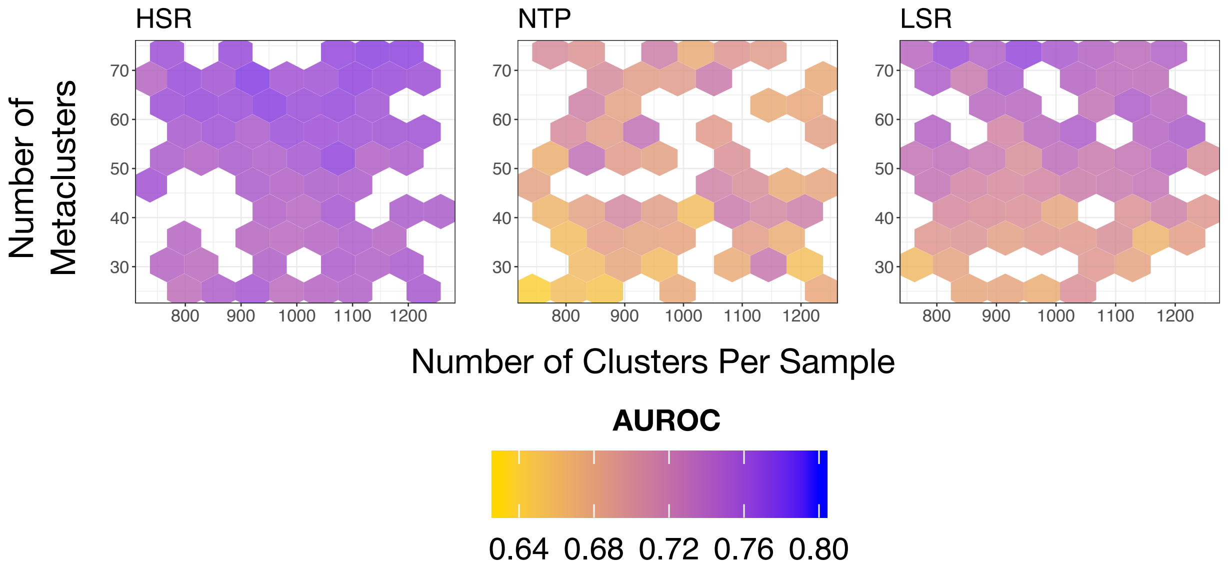

Supplementary Figure 18: **The Effect of VoPo Parameters on Classification Accuracy.** We explored the interplay between the number of within-sample clusters and number of between-sample metaclusters. Over 100 trials, values for these two parameters were selected from between 750 and 1250 for the number of within-sample clusters and from between 25-75 as the number of metaclusters. VoPo was run with these selected parameters classification accuracy as area under the ROC curver (AUROCC) was recorded. Classification accuracy as a function of these two parameters is visualized in a 2D plot with hexagonal bins. The color of each bin represents the AUC of the associated binned points, with orange (blue) indicating lower (higher) classification accuracy.

Choosing the Number of Per Sample Clusters and Across Sample Metaclusters

VoPo has two main free parameters, corresponding to the number of within-sample clusters and across-sample metaclusters. To understand the relationship between these two parameters, we set up an experiment for each dataset where over 100 trials, we randomly generated a pair of parameters for the number of within-sample clusters and across-sample metaclusters. The number of within-sample clusters was chosen randomly and uniformly between 750 and 1250 and the number of metaclusters was chosen randomly and uniformly between 25 and 75. For each of the 100 trials, VoPo was run with these selected parameters and the area under the ROC curve (AUC) was recorded. As this was not a full grid search, we sought to visualize general patterns about the interplay between these two parameters. To visualize such patterns, we used hexagonal binning to visualize the results (Supplementary Figure 18), where each bin is colored by the mean AUC of the associated binned points. These plots suggest that the choice of and combination of these parameters does not cause the AUC to vary significantly. Therefore for consistency, we selected 1,000 per-sample clusters and 50 metaclusters as our default parameters. These parameters were used for all experiments.

In practice, VoPo is a modular framework and can be applied with a user's choice and combination of clustering, metaclustering, feature selection, and classification approaches. Each of these components have a varying number of free parameters and should be optimized using a two-layer cross validation procedure.

Understanding Cell Populations Differing Between Clinical Phenotype Classes

We further performed additional analysis to understand the significant populations prioritized by VoPo. In each of the three datasets, we identified two cell-populations per dataset with notable differentiation scores between groups. For each of these studied cell-populations, we extracted the corresponding cell frequencies of samples from each clinical phenotype group and plotted their distributions as boxplots (Supplementary Fig 19). Here, we plot normalized frequencies for each of the shown cell-populations. Each frequency was normalized to the total number of mononuclear cells. Our analysis reveals that cell-populations prioritized by VoPo are indeed different between clinical phenotype classes.

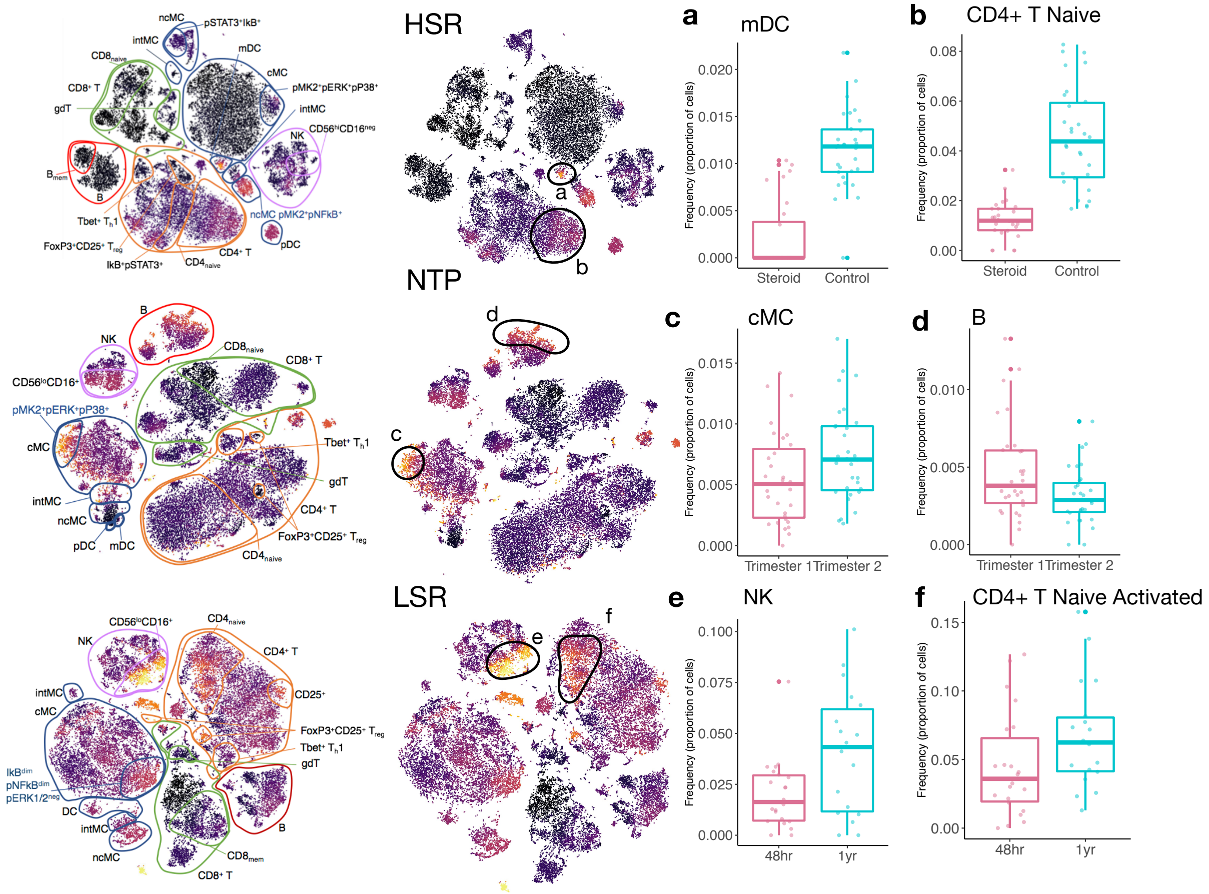

Supplementary Figure 19: **Examining Significant Cell-Populations Prioritized by VoPo.** Cell populations (or metaclusters) prioritized by VoPo were identified and distributions of the corresponding cell-frequencies between clinical phenotype classes were visualized. **a-b** Pink and turquoise boxplots show the frequencies of mDCs and $CD4^+$ naive T cells, respectively. **c-d** Pink and turquoise boxplots show the frequencies of cMCs and B cells of Trimester 1 and Trimester 2 samples, respectively. **e-f** Pink and turquoise boxplots show the frequencies of NK cells and $CD4^+$ T Naive activated cells in 48hr and 1yr samples, respectively. Our results suggest that VoPo does indeed identify cell-populations that differ between clinical phenotype classes. The boxplots show median values, interquartile range, whiskers of 1.5 times interquartile range, and all individual points.

As an alternative visualization to understand the cell-populations of interest, we used GateFinder [15] to automatically determine the combinations of markers that can be used to characterize the cell-populations of interest, according to VoPo. Briefly, we asked GateFinder to learn a two-step gating hierarchy. As a result, the algorithm identified marker combinations that can sequentially be used to identify the cells belonging to our populations of interest. These results are shown in Supplementary Fig 20. In the HSR dataset, our prioritized cell-populations correspond to mDCs (**a**) and naive $CD4^+$ T-cells (**b**). In the NTP dataset, we identified a subset of classical monocytes high in both CCR2 and CCR3 (**c**). In addition, we prioritized a B-cell subset expressing

HLADR, CXCR3, and CD45RA (**d**). Finally, in the LSR dataset, our GateFinder analysis shows that the first of our VoPo prioritized populations expresses CD16, Tbet, CD33, and CD45RA (**e**), together with a subpopulation of naive CD4$^+$ T cells (**f**).

In practice, using GateFinder to understand a subset of points prioritized by VoPo enables a cross-check between the original raw data and the VoPo clustering result. We suggest that a user identify a subset of cells of interest and input them into GateFinder for subsequent analysis. This will facilitate efficient understanding of the likely phenotype of a cell-population of interest.

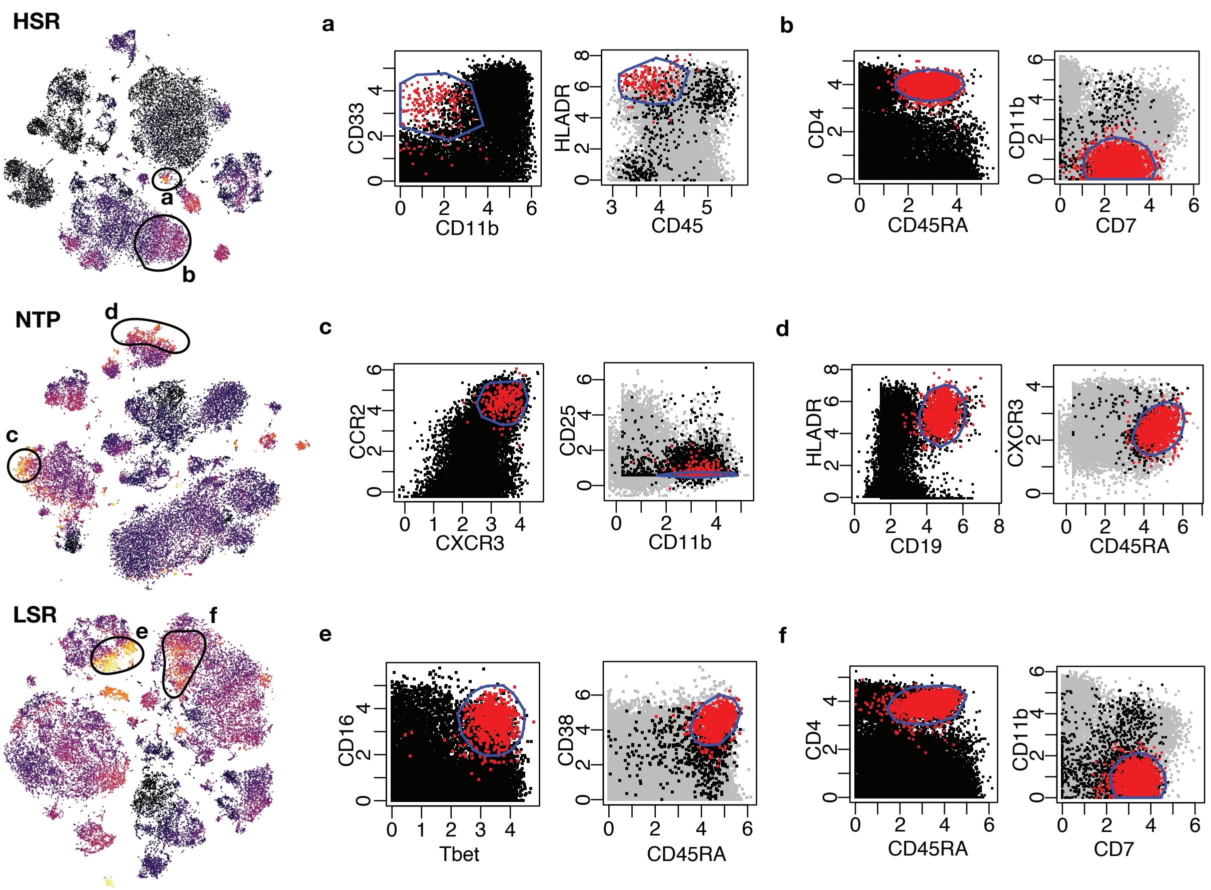

Supplementary Figure 20: **GateFinder Analysis of Cell-Populations Prioritized by VoPo.** Cell populations (or metaclusters) prioritized by VoPo were identified and GateFinder [15] was used to automatically determine the associated combination of markers and two-step hierarchical gating strategy. For each dataset, two example subsets of cells were selected and input into GateFinder and the associated two-step gating strategy was plotted (**a-f**). Selected cells are marked in red in panels **a-f**.

Supplementary Methods

Mass Cytometry Methods

Mass cytometry is a high-parameter single-cell analysis platform that enables the simultaneous interrogation of multiple signaling pathways in precisely phenotyped cell subsets spanning the entire immune system [16]. This technology provides unprecedented opportunities to describe the human immune system as a network of correlated, cell type-specific attributes and to investigate the functional relationships between cells within and across hematopoietic lineages [17, 18]. In Supplementary Tables 1-3 we provide a list of antibodies used in the HSR, NTP, and LSR datasets, respectively.

In Supplementary Figure we show the gating strategy used for each of the clinical datasets. The names of cell populations in blue were gated across all three datasets. Conversely, cell populations colored orange and green represent populations that were only identified in the HSR or LSR datasets, respectively (See chart).

Isotope	Antibody	Clone	Antibody Source	Target marker	Concentration	Cat.No.
102Pd	Barcode 1	na	Trace Sciences	Barcoding	15μM	Custom
104Pd	Barcode 2	na	Trace Sciences	Barcoding	15μM	Custom
105Pd	Barcode 3	na	Trace Sciences	Barcoding	15μM	Custom
106Pd	Barcode 4	na	Trace Sciences	Barcoding	15μM	Custom
108Pd	Barcode 5	na	Trace Sciences	Barcoding	15μM	Custom
110Pd	Barcode 6	na	Trace Sciences	Barcoding	15μM	Custom
113In	CD235ab	HIR2	Biolegend	Phenotype	1μg/mL	306615
113In	CD61	VI-PL2	BD	Phenotype	0.5μg/mL	555752
115In	CD45	HI30	Biolegend	Phenotype	1μg/mL	304045
139La	CD66	CD66a-B1.1	BD	Phenotype	0.5μg/mL	551354
141Pr	CD7	M-T701	BD	Phenotype	0.5μg/mL	555359
142Nd	CD19	HIB19	Biolegend	Phenotype	0.5μg/mL	302247
143Nd	CD45RA	HI100	Biolegend	Phenotype	0.5μg/mL	304143
144Nd	CD11b	ICRF44	Biolegend	Phenotype	2μg/mL	301337
145Nd	CD4	RPA-T4	Biolegend	Phenotype	2μg/mL	300541
146Nd	CD8a	RPA-T8	Biolegend	Phenotype	1μg/mL	557084
147Sm	CD11c	Bu15	Biolegend	Phenotype	1μg/mL	337221
148Nd	CD123	6H6	Biolegend	Phenotype	1μg/mL	306027
149Sm	pCREB (pS133)	87G3	Cell Signaling Technology	Function	2μg/mL	9198 (custom)
150Nd	pSTAT5 (pY694)	C11C5	Cell Signaling Technology	Function	4μg/mL	9359 (custom)
151Eu	pP38 (pT180/pY182)	36/p38	BD	Function	2μg/mL	612281 (custom)
152Sm	TCR__	B1	BD	Phenotype	4μg/mL	555715
153Eu	pSTAT1 (pY701)	14/P-STAT1	BD	Function	1μg/mL	612132 (custom)
154Sm	pSTAT3 (pY705)	M9C6	Cell Signaling Technology	Function	2μg/mL	4113 (custom)
155Gd	prpS6 (pS235/pS236)	D57.2.2E	Cell Signaling Technology	Function	2μg/mL	4858 (custom)
156Gd	CD24	ML5	Biolegend	Phenotype	2μg/mL	311127
157Gd	CD161	HP-3G10	Biolegend	Phenotype	2μg/mL	339919
158Gd	CD33	WM53	Biolegend	Phenotype	2μg/mL	303419
159Tb	pMAPKAPK2 (pT334)	27B7	Cell Signaling Technology	Function	1μg/mL	3007 (custom)
160Gd	Tbet	4B10	Thermo Fisher	Phenotype	8μg/mL	14-5825-82
161Dy	cPARP	F21-852	BD	Live/Dead	1μg/mL	552597
162Dy	FoxP3	PCH101	Thermo Fisher	Phenotype	8μg/mL	14-4776-82
164Dy	I_B	L35A5	Cell Signaling Technology	Function	8μg/mL	4814 (custom)
165Ho	CD16	3G8	Biolegend	Phenotype	1μg/mL	302051
166Er	pNFkB (pS529)	K10-895.12.50	BD	Function	2μg/mL	558393
167Er	pERK1/2 (pT202/pY204)	D13.14.4E	Cell Signaling Technology	Function	4μg/mL	4370 (custom)
168Er	pSTAT6	A15137E	Biolegend	Function	1μg/mL	686002
169Tm	CD25	M-A251	Biolegend	Phenotype	2μg/mL	356102
170Er	CD3	UCHT1	Biolegend	Phenotype	1μg/mL	300443
171Yb	CD27	M-T271	BD	Phenotype	2μg/mL	555439
172Yb	CD15	W6D3	Biolegend	Phenotype	8μg/mL	323035
173Yb	CCR2	K036C2	Biolegend	Phenotype	2μg/mL	357202
174Yb	HLA-DR	L243	Fluidigm	Phenotype	2μg/mL	3174001B
175Lu	CD14	M5E2	Fluidigm	Phenotype	2μg/mL	3175015B
176Lu	CD56	NCAM16.2	Biolegend	Phenotype	1μg/mL	318345
191Ir	DNA1	na	Fluidigm	Cell verification	50μM	201192A
193Ir	DNA2	na	Fluidigm	Cell verification	50μM	201192A

Supplementary Table 1: Antibody Panel in the HSR Dataset

Isotope	Antibody	Antibody Clone	Isotope/Antibody Source	Target marker	Concentration	Cat.No.
102Pd	Barcode 1	na	Trace Sciences	Barcoding	15μM	Custom
104Pd	Barcode 2	na	Trace Sciences	Barcoding	15μM	Custom
105Pd	Barcode 3	na	Trace Sciences	Barcoding	15μM	Custom
106Pd	Barcode 4	na	Trace Sciences	Barcoding	15μM	Custom
108Pd	Barcode 5	na	Trace Sciences	Barcoding	15μM	Custom
110Pd	Barcode 6	na	Trace Sciences	Barcoding	15μM	Custom
113In	CD235ab	HIR2	Biolegend	Phenotype	1μg/mL	306615
113In	CD61	VI-PL2	BD	Phenotype	0.5μg/mL	555752
115In	CD45	HI30	Biolegend	Phenotype	1μg/mL	304045
139La	CD66	CD66a-B1.1	BD	Phenotype	0.5μg/mL	551354
141Pr	CD7	M-T701	BD	Phenotype	0.5μg/mL	555359
142Nd	CD19	HIB19	Biolegend	Phenotype	0.5μg/mL	302247
143Nd	CD45RA	HI100	Biolegend	Phenotype	0.5μg/mL	304143
144Nd	CD11b	ICRF44	Biolegend	Phenotype	2μg/mL	301337
145Nd	CD4	RPA-T4	Biolegend	Phenotype	2μg/mL	300541
146Nd	CD8a	RPA-T8	Biolegend	Phenotype	1μg/mL	557084
147Sm	CD11c	Bu15	Biolegend	Phenotype	1μg/mL	337221
148Nd	CD123	6H6	Biolegend	Phenotype	1μg/mL	306027
149Sm	pCREB (pS133)	87G3	Cell Signaling Technology	Function	2μg/mL	9198 (custom)
150Nd	pSTAT5 (pY694)	47	Cell Signaling Technology	Function	4μg/mL	9359 (custom)
151Eu	pP38 (pT180/pY182)	36/p38/pT18	BD	Function	2μg/mL	612281 (custom)
152Sm	TCRgd	GL3	BD	Phenotype	4μg/mL	555715
153Eu	pSTAT1 (pY701)	58D6	BD	Function	1μg/mL	612132 (custom)
154Sm	pSTAT3 (pY705)	4/P pY705	Cell Signaling Technology	Function	2μg/mL	4113 (custom)
155Gd	prpS6 (pS235/pS236)	N7-548	Cell Signaling Technology	Function	2μg/mL	4858 (custom)
158Gd	CD33	WM53	Biolegend	Phenotype	2μg/mL	303419
159Tb	pMAPKAPK2 (pT334)	27B7	Cell Signaling Technology	Function	1μg/mL	3007 (custom)
160Gd	Tbet	4B10	Thermo Fisher	Phenotype	8μg/mL	14-5825-82
162Dy	FoxP3	PCH101	Thermo Fisher	Phenotype	8μg/mL	14-4776-82
164Dy	IkB	L35A5	Cell Signaling Technology	Function	8μg/mL	4814 (custom)
165Ho	CD16	3G8	Biolegend	Phenotype	1μg/mL	302051
166Er	pNFkB (pS529)	K10-895.12.50	BD	Function	2μg/mL	558393
167Er	pERK1/2 (pT202/pY204)	D13.14.4E	Cell Signaling Technology	Function	4μg/mL	4370 (custom)
169Tm	CD25	2A3	Biolegend	Phenotype	2μg/mL	356102
170Er	CD3	UCHT1	Biolegend	Phenotype	1μg/mL	300443
172Yb	CD15	W6D3	Biolegend	Phenotype	8μg/mL	323035
174Yb	HLA-DR	L243	Fluidigm	Phenotype	2μg/mL	3174001B
175Lu	CD14	M52E	Fluidigm	Phenotype	2μg/mL	3175015B
176Lu	CD56	NCAM16.2	Biolegend	Phenotype	1μg/mL	318345
191Ir	DNA1	na	Fluidigm	Cell verification	50μM	201192A
193Ir	DNA2	na	Fluidigm	Cell verification	50μM	201192A

Supplementary Table 2: Antibody Panel in the NTP Dataset

Isotope	Antibody	Antibody Clone	Antibody Source	Target marker	Concentration	Cat.No.
102Pd	Barcode 1	na	Trace Sciences	Barcoding	15μM	Custom
104Pd	Barcode 2	na	Trace Sciences	Barcoding	15μM	Custom
105Pd	Barcode 3	na	Trace Sciences	Barcoding	15μM	Custom
106Pd	Barcode 4	na	Trace Sciences	Barcoding	15μM	Custom
108Pd	Barcode 5	na	Trace Sciences	Barcoding	15μM	Custom
110Pd	Barcode 6	na	Trace Sciences	Barcoding	15μM	Custom
113In	CD235ab	HIR2	Biolegend	Phenotype	1μg/mL	306615
113In	CD61	VI-PL2	BD	Phenotype	0.5μg/mL	555752
115In	CD45	HI30	Biolegend	Phenotype	1μg/mL	304045
139La	CD66	CD66a-B1.1	BD	Phenotype	0.5μg/mL	551354
141Pr	CD7	M-T701	BD	Phenotype	0.5μg/mL	555359
142Nd	CD19	HIB19	Biolegend	Phenotype	0.5μg/mL	302247
143Nd	CD45RA	HI100	Biolegend	Phenotype	0.5μg/mL	304143
144Nd	CD11b	ICRF44	Biolegend	Phenotype	2μg/mL	301337
145Nd	CD4	RPA-T4	Biolegend	Phenotype	2μg/mL	300541
146Nd	CD8a	RPA-T8	Biolegend	Phenotype	1μg/mL	557084
147Sm	CD11c	Bu15	Biolegend	Phenotype	1μg/mL	337221
148Nd	CD123	6H6	Biolegend	Phenotype	1μg/mL	306027
149Sm	pCREB (pS133)	87G3	Cell Signaling Technology	Function	2μg/mL	9198 (custom)
150Nd	pSTAT5 (pY694)	47	Cell Signaling Technology	Function	4μg/mL	9359 (custom)
151Eu	pP38 (pT180/pY182)	36/p38/pT18	BD	Function	2μg/mL	612281 (custom)
152Sm	TCRgd	GL3	BD	Phenotype	4μg/mL	555715
153Eu	pSTAT1 (pY701)	58D6	BD	Function	1μg/mL	612132 (custom)
154Sm	pSTAT3 (pY705)	4/P pY705	Cell Signaling Technology	Function	2μg/mL	4113 (custom)
155Gd	prpS6 (pS235/pS236)	N7-548	Cell Signaling Technology	Function	2μg/mL	4858 (custom)
156Gd	CD24	ML5	Biolegend	Phenotype	2μg/mL	311127
157Gd	CD38	HIT2	Biolegend	Phenotype	4μg/mL	303535
158Gd	CD33	WM53	Biolegend	Phenotype	2μg/mL	303419
159Tb	pMAPKAPK2 (pT334)	27B7	Cell Signaling Technology	Function	1μg/mL	3007 (custom)
160Gd	Tbet	4B10	Thermo Fisher	Phenotype	8μg/mL	14-5825-82
161Dy	cPARP	F21-852	BD	Live/Dead	1μg/mL	552597
162Dy	FoxP3	PCH101	Thermo Fisher	Phenotype	8μg/mL	14-4776-82
164Dy	I_B	L35A5	Cell Signaling Technology	Function	8μg/mL	4814 (custom)
165Ho	CD16	3G8	Biolegend	Phenotype	1μg/mL	302051
166Er	pNFkB (pS529)	K10-895.12.50	BD	Function	2μg/mL	558393
167Er	pERK1/2 (pT202/pY204)	D13.14.4E	Cell Signaling Technology	Function	4μg/mL	4370 (custom)
168Er	pSTAT6	A15137E	Biolegend	Function	1μg/mL	686002
169Tm	CD25	M-A251	Biolegend	Phenotype	2μg/mL	356102
170Er	CD3	UCHT1	Biolegend	Phenotype	1μg/mL	300443
171Yb	CD27	M-T271	BD	Phenotype	2μg/mL	555439
172Yb	IgM	MHM-88	Fluidigm	Phenotype	4μg/mL	3172004B
173Yb	CCR2	K036C2	Biolegend	Phenotype	2μg/mL	357202
174Yb	HLA-DR	L243	Fluidigm	Phenotype	2μg/mL	3174001B
175Lu	CD14	M5E2	Fluidigm	Phenotype	2μg/mL	3175015B
176Lu	CD56	NCAM16.2	Biolegend	Phenotype	1μg/mL	318345
191Ir	DNA1	na	Fluidigm	Cell verification	50μM	201192A
193Ir	DNA2	na	Fluidigm	Cell verification	50μM	201192A

Supplementary Table 3: Antibody Panel in the LSR Dataset

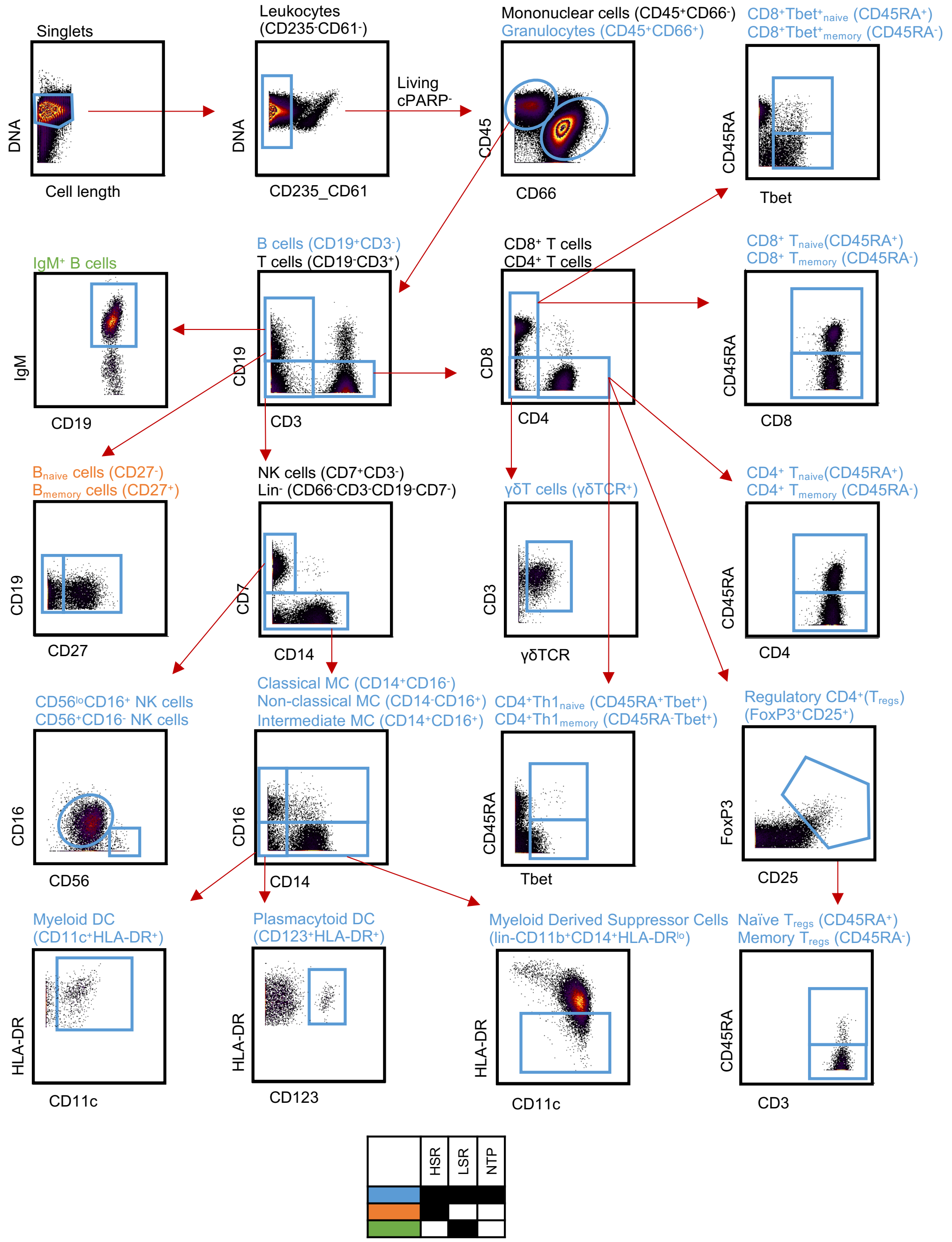

Supplementary Figure 21: **Gating Strategies in Original Analyses of HSR, NTP, and LSR.** Gating Strategies Used in Each Clinical Dataset in the Original Analysis. A different gating strategy was used for each of the three clinical datasets. Cell-populations in blue are common to all three clinical datasets. Cell types labeled orange and green represent populations that were only identified in the HSR or LSR datasets, respectively (See Supplementary Tables 1-3.). For example, only IgM^+ B-cells were gated in the LSR dataset and $CD27^+$ memory B-cells were gated only in the HSR dataset. In the VoPo pipeline, singlet live leukocytes ($DNA^+cPARP^-CD235^-CD61^-$) were used (Figure 2, Supplementary Figures 6–12, 14–16, 19, 20)

Supplementary References

[1] Bruggner, R. V., Bodenmiller, B., Dill, D. L., Tibshirani, R. J. & Nolan, G. P. Automated identification of stratifying signatures in cellular subpopulations. *Proceedings of the National Academy of Sciences* **111**, E2770–E2777 (2014).

[2] Qiu, P. *et al.* Extracting a cellular hierarchy from high-dimensional cytometry data with spade. *Nature biotechnology* **29**, 886 (2011).

[3] Hu, Z., Glicksberg, B. S. & Butte, A. J. Robust prediction of clinical outcomes using cytometry data. *Bioinformatics* **35**, 1197–1203 (2018).

[4] Van Gassen, S. *et al.* Flowsom: Using self-organizing maps for visualization and interpretation of cytometry data. *Cytometry Part A* **87**, 636–645 (2015).

[5] Levine, J. H. *et al.* Data-driven phenotypic dissection of aml reveals progenitor-like cells that correlate with prognosis. *Cell* **162**, 184–197 (2015).

[6] Tibshirani, R. Regression shrinkage and selection via the lasso. *Journal of the Royal Statistical Society: Series B (Methodological)* **58**, 267–288 (1996).

[7] Maaten, L. v. d. & Hinton, G. Visualizing data using t-sne. *Journal of machine learning research* **9**, 2579–2605 (2008).

[8] Lun, A. T., Richard, A. C. & Marioni, J. C. Testing for differential abundance in mass cytometry data. *Nature methods* **14**, 707 (2017).

[9] Tang, J., Liu, J., Zhang, M. & Mei, Q. Visualizing large-scale and high-dimensional data. In *Proceedings of the 25th international conference on world wide web*, 287–297 (International World Wide Web Conferences Steering Committee, 2016).

[10] Aghaeepour, N. *et al.* An immune clock of human pregnancy. *Science immunology* **2**, eaan2946 (2017).

[11] Watanabe, M. *et al.* Changes in t, b, and nk lymphocyte subsets during and after normal pregnancy. *American Journal of Reproductive Immunology* **37**, 368–377 (1997).

[12] Kraus, T. A. *et al.* Characterizing the pregnancy immune phenotype: results of the viral immunity and pregnancy (vip) study. *Journal of clinical immunology* **32**, 300–311 (2012).

[13] Joachims, T. Transductive learning via spectral graph partitioning. In *Proceedings of the 20th International Conference on Machine Learning (ICML-03)*, 290–297 (2003).

[14] Stanescu, A., Tangirala, K. & Caragea, D. Study of transductive learning and unsupervised feature construction methods for biological sequence classification. In *2016 IEEE/ACM International Conference on Advances in Social Networks Analysis and Mining (ASONAM)*, 999–1006 (IEEE, 2016).

[15] Aghaeepour, N. *et al.* Gatefinder: projection-based gating strategy optimization for flow and mass cytometry. *Bioinformatics* **34**, 4131–4133 (2018).

[16] Bendall, S. C. & Nolan, G. P. From single cells to deep phenotypes in cancer. *Nature biotechnology* **30**, 639 (2012).

[17] Gaudillière, B. *et al.* Implementing mass cytometry at the bedside to study the immunological basis of human diseases: distinctive immune features in patients with a history of term or preterm birth. *Cytometry Part A* **87**, 817–829 (2015).

[18] Spitzer, M. H. *et al.* Systemic immunity is required for effective cancer immunotherapy. *Cell* **168**, 487–502 (2017).
