# Supplementary material for: VoPo leverages cellular heterogeneity for predictive modeling of single-cell data
Source: Nat Commun. 2020 Jul 27;11:3738. doi: 10.1038/s41467-020-17569-8 (PMC7385162; doi:10.1038/s41467-020-17569-8)
Supplement: Supplementary file 2 — Peer Review File [file 41467_2020_17569_MOESM2_ESM.pdf]

nature research

Corresponding author(s): Nima Aghaeepour

Last updated by author(s): June 29, 2020

Reporting Summary

Nature Research wishes to improve the reproducibility of the work that we publish. This form provides structure for consistency and transparency in reporting. For further information on Nature Research policies, see our Editorial Policies and the Editorial Policy Checklist.

Statistics

For all statistical analyses, confirm that the following items are present in the figure legend, table legend, main text, or Methods section.

n/a	Confirmed	
☐	☒	The exact sample size (*n*) for each experimental group/condition, given as a discrete number and unit of measurement
☐	☒	A statement on whether measurements were taken from distinct samples or whether the same sample was measured repeatedly
☐	☒	The statistical test(s) used AND whether they are one- or two-sided<br>*Only common tests should be described solely by name; describe more complex techniques in the Methods section.*
☒	☐	A description of all covariates tested
☒	☐	A description of any assumptions or corrections, such as tests of normality and adjustment for multiple comparisons
☐	☒	A full description of the statistical parameters including central tendency (e.g. means) or other basic estimates (e.g. regression coefficient) AND variation (e.g. standard deviation) or associated estimates of uncertainty (e.g. confidence intervals)
☐	☒	For null hypothesis testing, the test statistic (e.g. F, t, r) with confidence intervals, effect sizes, degrees of freedom and P value noted<br>*Give P values as exact values whenever suitable.*
☒	☐	For Bayesian analysis, information on the choice of priors and Markov chain Monte Carlo settings
☒	☐	For hierarchical and complex designs, identification of the appropriate level for tests and full reporting of outcomes
☒	☐	Estimates of effect sizes (e.g. Cohen's *d*, Pearson's *r*), indicating how they were calculated

Our web collection on statistics for biologists contains articles on many of the points above.

Software and code

Policy information about availability of computer code

Data collection	All datasets used are publicly available through flowrepository under experiment IDs FR-FCM-ZY3Q, FR-FCM-ZY3R, FR-FCM-Z2AT, and FR-FCM-ZYSB for the NTP Training, NTP Validation, HSR, and LSR datasets, respectively.
Data analysis	VoPo was implemented and tested in R version 3.4.4. Code to reproduce the results is available in the following link. (https://nalab.stanford.edu/vopo/)

For manuscripts utilizing custom algorithms or software that are central to the research but not yet described in published literature, software must be made available to editors and reviewers. We strongly encourage code deposition in a community repository (e.g. GitHub). See the Nature Research guidelines for submitting code & software for further information.

Data

Policy information about availability of data

All manuscripts must include a data availability statement. This statement should provide the following information, where applicable:

- Accession codes, unique identifiers, or web links for publicly available datasets
- A list of figures that have associated raw data
- A description of any restrictions on data availability

Mass cytometry data have been made publicly available in the flow repository: HSR data set: FR-FCM-Z2AT; NTP: FR-FCMZY3Q, FR-FCM-ZY3R; LSR: FR-FCM-ZYSB

Field-specific reporting

Please select the one below that is the best fit for your research. If you are not sure, read the appropriate sections before making your selection.

☒ Life sciences ☐ Behavioural & social sciences ☐ Ecological, evolutionary & environmental sciences

For a reference copy of the document with all sections, see nature.com/documents/nr-reporting-summary-flat.pdf

Life sciences study design

All studies must disclose on these points even when the disclosure is negative.

Sample size	Sample size determination was conducted for each of the three independent, previously published studies. Please refer to the original publication of each study.
Data exclusions	Data exclusion, if any, was conducted for each of the three independent, previously published studies. Please refer to the original publication of each study.
Replication	Measures of reproducibility were conducted for each of the three independent, previously published studies. Please refer to the original publication of each study.
Randomization	Samples were grouped according to the study objective in each of the three independent, previously published studies. Please refer to the original publication of each study.
Blinding	Blinding, if any, was conducted for each of the three independent, previously published studies. Please refer to the original publication of each study.

Reporting for specific materials, systems and methods

We require information from authors about some types of materials, experimental systems and methods used in many studies. Here, indicate whether each material, system or method listed is relevant to your study. If you are not sure if a list item applies to your research, read the appropriate section before selecting a response.

Materials & experimental systems

n/a	Involved in the study	
☐	☒	Antibodies
☒	☐	Eukaryotic cell lines
☒	☐	Palaeontology and archaeology
☒	☐	Animals and other organisms
☐	☒	Human research participants
☒	☐	Clinical data
☒	☐	Dual use research of concern

Methods

n/a	Involved in the study	
☒	☐	ChIP-seq
☐	☒	Flow cytometry
☒	☐	MRI-based neuroimaging

Antibodies

Antibodies used	For antibodies used, please refer to the original publications of the studies used in this publication. Please also refer to the list of antibodies in the supplement of this publication.
Validation	Please see above.

Human research participants

Policy information about studies involving human research participants

Population characteristics	A table with all demographic information associated with each data set has been published in the original publication of each study.
Recruitment	Please refer to the original publications of each of the three data sets.
Ethics oversight	Stanford University Institutional Review Board (IRB)

Note that full information on the approval of the study protocol must also be provided in the manuscript.

Flow Cytometry

Plots

Confirm that:

- [x] The axis labels state the marker and fluorochrome used (e.g. CD4-FITC).
- [ ] The axis scales are clearly visible. Include numbers along axes only for bottom left plot of group (a 'group' is an analysis of identical markers).
- [ ] All plots are contour plots with outliers or pseudocolor plots.
- [ ] A numerical value for number of cells or percentage (with statistics) is provided.

Methodology

Sample preparation	Please refer to the original publications of each of the three data sets. In short, a whole-blood immunoassay was performed, followed by sample barcoding, antibody staining, and mass cytometry measurement. Measures to minimize the effect of experimental variability on mass cytometry measurements were implemented. Immune features (live, singlet cells) were extracted for downstream analyses.
Instrument	Helios Mass Cytometer (Fluidigm Inc., South San Francisco, CA, USA)
Software	The mass cytometry data was normalized using Normalizer v0.1 MATLAB Compiler Runtime (MathWorks). Files were then de-barcoded with a single-cell MATLAB debarcoding tool. Manual gating was performed using CellEngine (https://immuneatlas.org/#/) (Primity Bio, Fremont, CA).
Cell population abundance	N/A
Gating strategy	The gating strategy that has been used in each of the three studies is provided in the supplement.

- [x] Tick this box to confirm that a figure exemplifying the gating strategy is provided in the Supplementary Information.
